# Mechanochemical Approach to a Monocationic Asymmetric Monomethine Cyanine Dye for Nucleic Acid Analysis and Visualization

**DOI:** 10.3390/molecules30193966

**Published:** 2025-10-02

**Authors:** Diana Cheshmedzhieva, Nadezhda Bozova, Sonia Ilieva, Christo Novakov, Aleksey Vasilev

**Affiliations:** 1 Faculty of Chemistry and Pharmacy, Sofia University “St. Kliment Ohridski”, 1 J. Bourchier Ave., 1164 Sofia, Bulgaria; nadegdabozova@abv.bg (N.B.); silieva@chem.uni-sofia.bg (S.I.); 2 Institute of Polymers, Bulgarian Academy of Sciences, Akad. G. Bonchev St., bl 103A, 1113 Sofia, Bulgaria; hnovakov@polymer.bas.bg

**Keywords:** monomethine cyanine dye, fluorogenic probes, RNA, DNA, biomolecular probes, aggregachromism

## Abstract

Using an environmentally friendly approach, we successfully synthesized an asymmetric monomethine cyanine dye, 7-chloro-1-ethyl-4-((3-ethylbenzo[d]thiazol-2(3H)-ylidene)methyl) quinolin-1-ium iodide, named CHLoris (CHL), via a modified Knoevenagel-type condensation. The reaction was carried out mechanochemically in an ethanol–water medium using 1-ethyl-2-methylbenzothiazolium iodide and 4,7-dichloro-1-ethylquinolin-1-ium iodide in the presence of sodium carbonate as a base and catalytic amounts of Hünig’s base. The UV/VIS absorption spectra of CHL in both the buffer solution and ethanol revealed the formation of aggregates in aqueous media. Density Functional Theory (DFT) and Time-Dependent DFT (TDDFT) calculations were employed to support the experimental findings further and provide insights into the self-association behavior of CHL in an aqueous solution. The photophysical properties of the dye were examined in the presence of DNA and RNA, and its performance was compared to that of the commercial dye Thiazole Orange (TO) under identical conditions. The results show that CHL is more sensitive towards RNA.

## 1. Introduction

**Figure 1:**
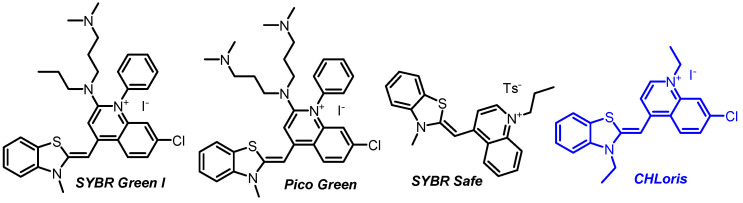
Chemical structures of the commercial dyes SYBR Green ITM, Pico GreenTM, SYBR SafeTM from the SYTO series (Thermo Fisher Scientific Inc., Waltham, MA, USA), and the newly synthesized analogue CHLoris (CHL).

Fluorescence is one of the main phenomena supporting biomedical analytical methods. A major proportion of the biomarkers used for the analysis of biological macromolecules and as reagents for labeling and visualizing cells, cell organelles, and cellular processes is fluorescence-generating substances [1,2,3,4,5,6,7,8]. These so-called fluorogenic labels (or “light-on” probes) are characterized by minimal or no intrinsic fluorescence in their unbound state [3]. When interacting with the target biological micro-objects, these compounds generate a powerful luminescent response that can be detected and quantified [3]. In addition, some of the mentioned substances have a pronounced affinity for certain types of biological macromolecules [9]. Such compounds are used as labeling agents for bimolecular recognition. Specifically, they are used to quantify and visualize DNA, RNA, proteins, cellular components, and more [1]. One of the main reagents for labeling biological macromolecules and nucleic acids in particular is the dye Ethidium bromide (EB). It has a number of advantages, such as high fluorescence quantum yield in the presence of nucleic acids, low cost, and good solubility in organic solvents and water. For these reasons, EB is one of the most widely applied dyes in DNA and RNA fluorescence analyses. Its inherent disadvantages, however, are its significant toxicity and carcinogenicity, which put the health of those working with this dye at serious risk [10]. An additional disadvantage is its high intrinsic fluorescence, which introduces a significantly high level of background fluorescent noise and reduces the sensitivity of analyses performed with this dye [10]. To address the previously mentioned limitations, scientists from the former American company Molecular Probes Inc. (Eugene, OR, USA), now part of the multinational corporation Thermo Fisher Scientific (Waltham, MA, USA), developed a series of highly successful dyes designed for labeling biological macromolecules [1]. These include the SYTO™ series—notably SYBR Green I, Pico Green, and SYBR Safe [1] (Figure 1). Due to their outstanding properties, such as low intrinsic fluorescence, high fluorescence quantum yield, low cytotoxicity, and non-carcinogenicity, these dyes have been widely adopted in biological research. For many years, however, they were marketed under proprietary code names, with their exact chemical structures kept undisclosed [1]. This proprietary information was revealed by two independent teams of German scientists. In 2004, the chemical structure of SYBR Green I was elucidated and published, followed by the disclosure of the SYBR Safe™ structure in 2008. Both dyes (Figure 1) are derivatives of Thiazole Orange (TO)—a well-known monomethine cyanine dye. TO belongs to the largest and most extensively studied class of nucleic acid labeling dyes. These dyes are indispensable tools for the visualization of DNA and RNA across a wide range of applications, including PCR techniques, fluorescence spectroscopy, flow cytometry, fluorescence intercalator displacement (FID) assays, and fluorescence in situ hybridization (FISH) [11,12].

Furthermore, the growing use of these dyes in various areas of biomedical analysis has led to an increased market demand. This, in turn, is associated with certain environmental requirements concerning the synthesis of such dyes. The most commonly used method for synthesizing dyes of this class is the so-called Brucker’s method. A disadvantage of the method is the release of foul-smelling methyl mercaptan. The remarkable properties of cyanine dyes have motivated us to continue our work in design and synthesis using environmentally friendly methods and the investigation of new analogues within this valuable class of compounds.

In recent years, mechanochemistry has become one of the preferred methods for the synthesis of organic compounds. This is due to the significant advantages of this type of promotion of chemical reactions, for example, the elimination of or drastic reduction in the organic solvents or the use of negligible amounts of them during synthesis. Another advantage is the acceleration of chemical reactions or reaching the activation energy for the processes with significantly fewer resources. The elimination of the need for heating chemical reactions leads to a reduction in energy costs. In general, it can be said the mechanochemistry as a green synthetic methodology has been fruitfully utilized in organic synthesis by using a mixer mill, a planetary ball mill, or a mortar and pestle [13]. In this regard, the aim of this study is to present new environmentally friendly synthetic conditions for the preparation of a novel TO analog and to investigate its photophysical properties both in its pure form in solution and in the presence of nucleic acids.

## 2. Results and Discussion

### 2.1. Synthesis

**Scheme 1:**
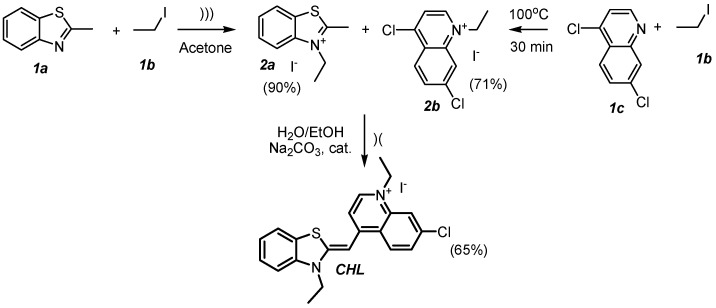
Synthetic pathway for the preparation of 7-chloro-1-ethyl-4-((3-ethylbenzo[d]thiazol-2(3H)-ylidene)methyl)quinolin-1-ium iodide (CHL).

The increasing application of asymmetric monomethine cyanine dyes such as TO in many areas of biomedical analysis raises the need to develop new, environmentally friendly synthetic procedures. The development of green synthetic methods for dye synthesis would enable their production in semi-industrial and industrial volumes. With these considerations in mind, we have developed an environmentally friendly synthetic procedure in which synthetic techniques consistent with the principles of green chemistry were used. Thus, the interaction between 2-methylbenzothiazole 1a with iodoethane 1b in acetone upon sonication in an ultrasonic bath leads to the formation of a white crystalline precipitate of 1-ethyl-2-methylbenzothiazolium iodide 2a (Scheme 1) in mild conditions. The chosen approach leads to the preparation of the pure product 2a directly from the reaction mixture without the need for further purification. The use of ultrasound reduces energy costs and accelerates the preparation of the desired product.

Following the principles of green chemistry, we performed the reaction of iodoethane 1b with 4,7-dichloroquinoline 1c in a high-pressure sealed tube without any solvent to obtain 4,7-dichloro-1-ethylquinolin-1-ium iodide (**2b**) in excellent yield (Scheme 1). Finally, we carried out the reaction of intermediates **2a** and **2b** by a mechanochemical method using sodium carbonate as a basic reagent, adding one drop of the Hünig’s base as an initiator. Replacing the standard amines such as triethylamine or DIPEA with sodium carbonate significantly reduces the cost of the synthesis of the target dye, while also having a less negative impact on the environment by avoiding the use of the aforementioned toxic and unpleasantly odorous reagents. Thus, in a modified Knoevenagel-type condensation reaction between intermediates **2a** and **2b**, we obtained the dye CHLoris (CHL) in mild conditions at 65% yield and with excellent purity. The advantages of the mechanochemical synthetic procedure presented in this work compared to classical methods are the following: 1. The dye is obtained at above-average yields. 2. No toxic solvents are used, and the amounts of solvents used are minimized. 3. We work with substances that do not emit toxic fumes. 4. The basic reagent DIPEA is reduced to catalytic amounts in exchange for the use of the more environmentally friendly and odorless sodium carbonate. 5. The reaction time is reduced to 30 min, unlike the other methods, where it is at least 2 h. A single recrystallization from ethanol allowed obtaining the final dye with analytical purity. The chemical structure of the dye was proven by ^1^H-NMR, ^13^C-NMR, DEPT, HSQC, HMBC, and MALDI-TOF mass spectrometry (see ESI). It should be noted that the results obtained from MALDI-TOF analyses performed both with the addition of a matrix (dithranol) and a cationizing salt and without them are identical. Obviously, the synthesized product is able to ionize and evaporate without the need of additional co-crystallization with a matrix. The type of spectrum and the data for the molecular mass of the newly synthesized compound depend primarily on the applied laser power and the operating mode used (reflectron or linear, see Scheme S11a,b). A higher resolution and mass accuracy is observed in reflectron mode, which is known as being more suitable for the analysis of low-molecular-weight compounds (Scheme S11a). The detailed view of the mass spectrum reveals the appearance of a certain number of satellite peaks to the main molecular ion peak (*m*/*z* = 367.4195), which can be attributed to the presence of sulfur isotopes in the heterocyclic moiety of the cyanine dye (see Scheme S11c,d). We also performed the reaction under conventional conditions previously reported by our group, designated as reference Procedure B (see Materials and Methods Section). While the yield of the target dye obtained by Procedure B (89%) was higher than that achieved with Procedure A (65%), this apparent advantage is outweighed by significant drawbacks. Procedure B requires a substantially larger amount of DIPEA—an expensive, malodorous, and toxic base—and relies on DCM, a highly hazardous solvent with well-documented environmental and health risks. Consequently, its applicability is limited to small-scale laboratory use. In contrast, the greener Procedure A, despite giving a somewhat lower yield, avoids these drawbacks and thus represents a far more practical and sustainable option for the synthesis of larger quantities of CHL or related analogues on an industrial or semi-industrial scale.

### 2.2. Photophysical Properties

#### 2.2.1. Photophysical Properties of the Neat Dye CHL

**Figure 2:**
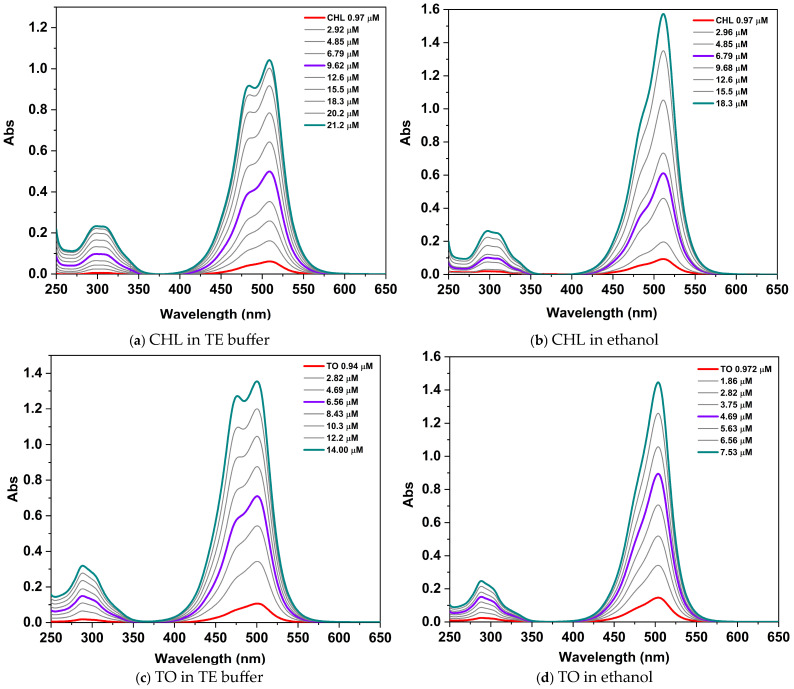
UV/VIS absorption spectra of CHL and TO at varying concentrations: (**a**) CHL in TE buffer, (**b**) CHL in ethanol, (**c**) TO in TE buffer, (**d**) TO in ethanol. The plots illustrate the dependence of absorption on dye concentration in different solvents.

The UV/VIS spectra of the dye CHL were recorded in ethanol and TE buffer (pH 8) (Figure 2). The absorption spectra of CHL at varying concentrations are presented in Figure 2a,b. At a low concentration (C_CHL_ = 9.7 × 10^−7^ M) in TE buffer (Figure 2a), only a single broad absorption band is detected in the visible region, with a maximum at 509 nm. As the dye concentration increases, the emergence of an additional absorption band with a maximum at 482 nm is observed. With an increasing concentration, the dye exhibits two overlapping absorption bands in the TE buffer, at 509 nm and 482 nm. The longer-wavelength band is attributed to dye monomers, while the shorter one corresponds to their aggregates (Figure 2a). In ethanol, the monomeric absorption peak is observed at 511 nm with a high intensity, while the aggregation band is low in intensity and appears as a shoulder of the high-intensity monomeric band. This behavior indicates reduced dye aggregation in the organic solvent (Figure 2b). The photophysical properties of the newly synthesized dye were compared with those of TO under the same experimental conditions (Figure 2c,d). The key spectral characteristics—λ_max_ and molar absorptivities (ε)—for both CHL and TO are summarized in Table 1. Notably, the presence of a chlorine atom in the quinoline ring of the newly synthesized dye induces a bathochromic shift in the absorption maximum.

The self-aggregation of asymmetric cyanine dyes in an aqueous environment is a well-known phenomenon [14]. These dyes can form various types of aggregates with different structural arrangements, depending on their molecular properties, as described in the literature [15,16]. For both CHL and TO, the absorption maxima observed in the range of 476–482 nm in an aqueous solution are attributed to dimer formation. These bands are blue-shifted by approximately 24–27 nm relative to the corresponding monomer bands (Table 1).

**Figure 3:**
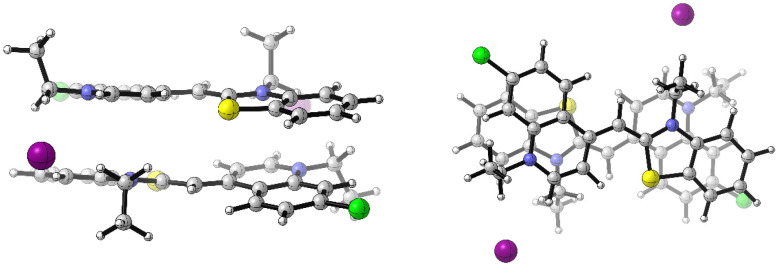
PCM/M062X/6-31G(d,p) optimized geometry of the most stable π-stacked H-dimer of the dye CHL in a water medium: left—side view, right—top view. Color scheme: O—purple, N—blue, S—yellow, C—gray, H—light gray [20].

Density Functional Theory (DFT) and Time-Dependent DFT (TDDFT) methods were employed to calculate the photophysical properties of the newly synthesized compounds and their aggregates. The geometries of the π-stacked dimers were optimized at the M062X/6-31G(d,p) level of theory in water medium, with the SDD basis set applied specifically to the iodide counterions [17,18,19]. A previous theoretical study clearly established that the E (trans) isomer is more stable, with the calculated energy difference between the cis and trans conformers of Thiazole Orange being 5.4 kcal/mol, in favor of the trans form [16]. The spatial arrangement of the most stable π-stacked dimer of CHL is shown in Figure 3, and the optimized geometry of the other H- and J-type dimers and their relative Gibbs free energies are given in ESI Figures S1 and S2. In the most stable H-type dimer, the donor part of the first CHL molecule is positioned above the acceptor part of the second CHL molecule (Figure 3). The predicted wavelengths of the vertical absorption transitions for the two dyes and their most stable dimers are summarized in Table 1. These predicted maxima, ranging from 471 to 474 nm, are blue-shifted relative to the calculated absorption maxima of the corresponding monomers (501–509 nm). The obtained theoretical results are in agreement with the experimental observations (Table 1) [15,16], supporting the formation of H-type dimers in an aqueous solution.

The Gibbs free energy of dimer formation for CHL was calculated and compared to that of TO and ID1. TO is the primary analogue of cyanine dyes, while ID1 is a chlorine-substituted dye that has been previously studied [21]. These calculations provide insights into the stability of the dye aggregates and support the experimental observations regarding self-association behavior in an aqueous environment. The Gibbs free energy of dimer formation for the three dyes was calculated using the M062X/6-31G(d,p) method based on the following model reaction: 2D → (D)_2_. The calculated Gibbs free energy for the formation of the H2 dimer of the dye CHL is −7.5 kcal/mol. The calculated Gibbs free energy of dimer formation for TO is −6.10 kcal/mol [8] and −5.6 kcal/mol for ID1.

In our previous study, excellent agreement was demonstrated between the experimentally determined dimerization constant K_D_ and theoretically computed Gibbs free energy (ΔG) for TO dimerization [16], which supports the accuracy of the computational model and validates the choice of the functional and the basis set. Based on the calculated Gibbs free energies for CHL, TO, and ID1 dimers, it can be concluded that CHL exhibits a greater aggregation propensity compared to TO and ID1.

#### 2.2.2. Photophysical Properties of CHL Dye Complexes with dsDNA and RNA

**Figure 4:**
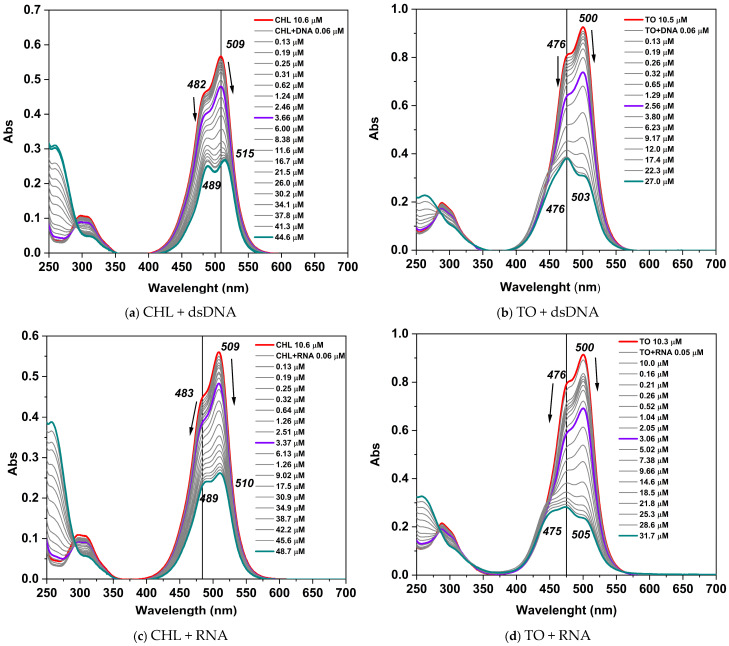
UV/VIS absorption of the dyes, neat and in the presence of increasing concentrations of nucleic acids, in TE buffer at pH 8: (**a**) CHL C = 10.6 µM and dsDNA; (**b**) TO C = 10.5 µM and dsDNA; (**c**) CHL C = 10.6 µM and RNA; (**d**) TO C = 10.3 µM and RNA. The arrows represent the direction of change in the absorbance of the dye–NA complexes as the nucleic acid concentration increases.

The interaction of CHL dye with dsDNA and RNA was investigated using UV/VIS and fluorescence spectroscopy, with the results compared to those obtained for TO. Changes in the absorption spectra of CHL and TO upon the addition of defined amounts of DNA and RNA are presented in Figure 4. The pronounced decrease in the intensity of the absorption maxima at 509 nm and 482 nm provides evidence of non-covalent dye–nucleic acid (NA) interactions and indicates the formation of dye–NA complexes. During the titration of CHL with DNA, a significant hypochromic effect is observed in the UV/VIS spectra—the absorbance at 509 nm decreases nearly two-fold, from 0.567 to 0.269 (Figure 4a). Both absorption peaks exhibit bathochromic and hypochromic shifts: the first absorption maximum shifts from 509 nm to 515 nm, and the second peak shifts from 482 nm to 488 nm. The red shift of the 509 nm band to the 515 nm is associated with the formation of monomer–NA complexes, while changes in the intensity of the 482 nm band are attributed to interactions involving dimeric dye aggregates. These spectral changes suggest the presence of two distinct binding modes: the bathochromic shift of the band evidences that monomeric dyes most likely interact through partial intercalation, whereas dimers bind within the minor groove.

Similar effects are observed during the titration of TO with dsDNA. A hypochromic effect is observed in the UV/VIS spectra—the absorption intensity decreases from 0.926 to 0.381. The main peak is at 500 nm. With the addition of DNA, this peak shows both a hypochromic and slight bathochromic shift (from 500 nm to 503 nm). The absorption maximum associated with the dimers exhibits a hypochromic shift but does not change its position (Figure 4b).

In the titration of CHL with RNA, a hypochromic effect is observed in the UV/VIS spectra—the absorption decreases approximately two-fold, from 0.560 to 0.262. The absorption maxima at 509 nm and 483 nm shift hypochromically but their position remains unchanged (Figure 4c). During the titration of TO with RNA, a hypochromic effect is observed in the UV/VIS spectra—the absorption decreases approximately three-fold, from 0.914 to 0.282. The main peak is at 500 nm and shifts bathochromically and hypochromically (from 500 nm to 505 nm—Figure 4d).

**Figure 5:**
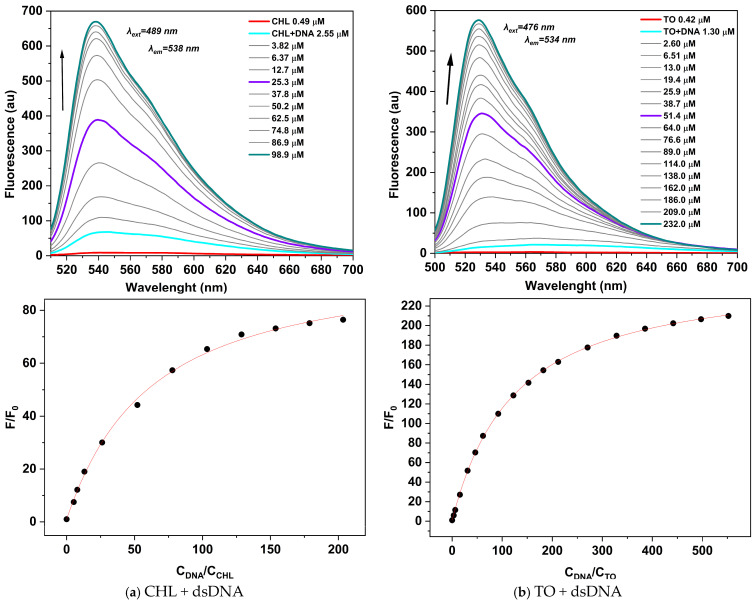

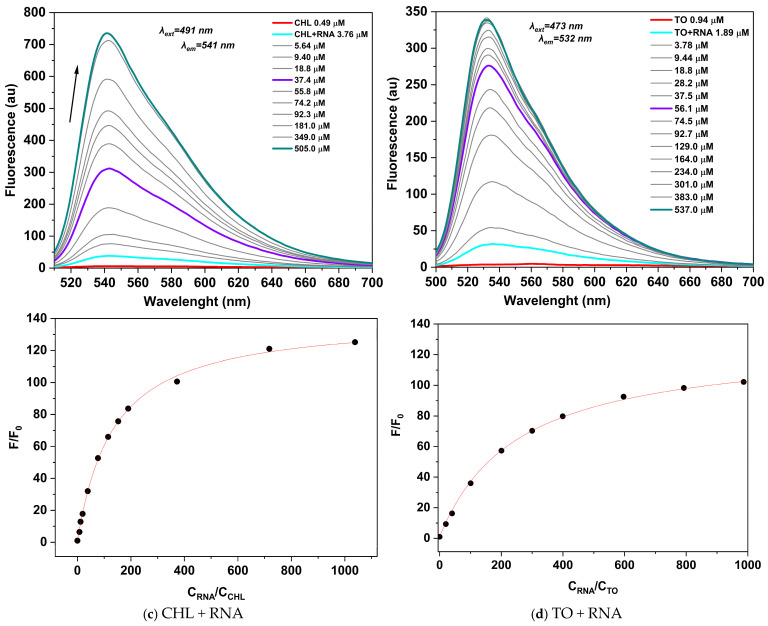
Fluorescence spectra of CHL and TO dyes in TE buffer and evolution of spectra upon addition of DNA and RNA. (**a**) CHL C = 0.49 µM and with dsDNA (start C_DNA_ = 3.19 mM) in TE buffer, λ_em_ = 538 nm of the complex, λ_ext_ = 489 nm; (**b**) TO (0.42 μM) with DNA (start C_DNA_ = 3.65 mM) in TE buffer, λ_em_ = 533 nm of the complex, λ_ext_ = 476 nm; (**c**) CHL C = 0.49 µM and with RNA (start C_RNA_ = 4.71 mM) in TE buffer, λ_em_ = 541 nm of the complex, λ_ext_ = 491 nm; (**d**) TO (0.09 μM) with RNA (start C_RNA_ = 4.73 mM) in TE buffer, λ_em_ = 532 nm of the complex, λ_ext_ = 473 nm.

Dye–nucleic acid interactions were further investigated using fluorescence spectroscopy. The key spectral characteristics of the dyes and their complexes with dsDNA and RNA are summarized in Table 2. The dyes were titrated with defined amounts of DNA and RNA, and the resulting changes in fluorescence spectra upon nucleic acid addition are presented in Figure 5. CHL and TO exhibit minimal intrinsic fluorescence in TE buffer; however, a significant increase in fluorescence intensity is observed upon binding to dsDNA or RNA. The initial concentrations of the dyes were 0.49 μM for CHL and 0.42 μM for TO.

Binding constants for each dye–nucleic acid complex were determined based on fluorescence titration experiments with dsDNA and RNA, as summarized in Table 2. In these experiments, a solution of dsDNA or RNA at a defined concentration was gradually added to a fixed concentration of the dye. After each addition, the mixture was incubated for 5 min to allow equilibrium to be reached before fluorescence measurements were taken. Titration data were analyzed according to the site-independent model through nonlinear fitting in the Origin 2023 software [24]. The resulting binding constant values (K_b_) indicate strong interactions with nucleic acids and suggest a partially intercalative mode of binding [15,25]. In our previous study on chlorine-substituted monomethine cyanine dyes (ID1 and ID2), a combination of analytical methods indicated the presence of multiple binding modes. It was shown that at low ligand-to-nucleotide binding ratios, intercalation is the dominant interaction mode, as demonstrated by FID assay experiments [21]. At higher binding ratios, groove-binding aggregates are formed, likely due to the hydrophobic nature of these dyes, which readily form dimers. The binding constant (K_b_) for TO with DNA, measured in this study, is in good agreement with values previously reported in Ref. [16] (lg K_b_ = 5.49). The newly synthesized dye CHL exhibits similar behavior to the dyes reported in Ref. [14].

**Figure 6:**
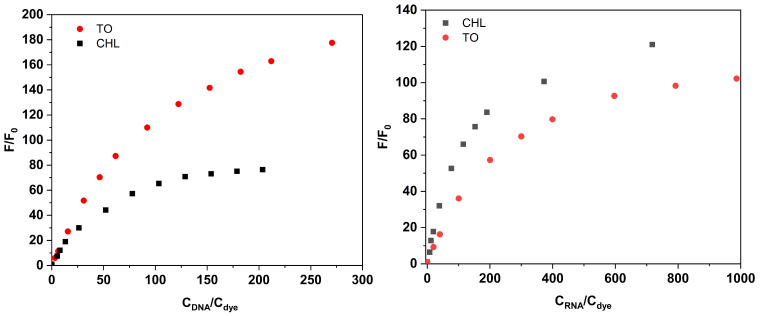
Changes in the fluorescence emission of CHL (C = 0.49 μM)) and TO (C = 0.42 μM) upon addition of dsDNA or RNA at pH 8 (TE buffer).

CHL demonstrates higher sensitivity toward RNA, with a 125-fold increase in fluorescence intensity upon RNA complexation compared to a 76-fold increase for DNA complexation (Table 2, Figure 6). This result aligns with previous observations that chlorine-substituted dyes exhibit higher sensitivity to RNA compared to dsDNA [21,26].

These findings clearly demonstrate that CHL is highly effective for nucleic acid staining and visualization.

## 3. Materials and Methods

### 3.1. General

The starting materials 1a, 1b, and 1c and the solvents reported in this work are commercial HPLC-grade products and were used as supplied. Melting points of the reaction products were measured on a Boetius PHMK 0.5 apparatus and are uncorrected. NMR spectra (^1^H-, ^13^C-NMR) were obtained on a Bruker Avance II + NMR spectrometer operating at 500 MHz for ^1^H- and 125 MHz for ^13^C-NMR in DMSO-*d*_6_ as a solvent. The chemical shifts are given in ppm (δ) using tetramethylsilane (TMS) as an internal standard. Matrix-assisted laser desorption/ionization–time of flight (MALDI-TOF) analyses were performed using a Shimadzu Axima Confidence TM mass spectrometer (Kratos Analytical Ltd., Stratford, UK) equipped with an ionization source working in positive or negative ion operation mode of variable ion extracting energy (+25kV/−20kV in linear; +20kV/−20kV in reflectron, resp.); a linear flight tube of 1.2 m drift length and reflectron 2.0 m drift length and detectors: linear mode—multiple dynodes and reflection mode—fast micro-channel plate. Experiments shown here were carried out both in linear and reflectron positive ion mode. Samples were prepared by dissolving the dye CHL (5 mg.mL^−1^) in ethanol–DMSO = 1:1 and premix with a matrix (mg.mL^−1^ dithranol in ethanol) and cationizing agent (0.1 mol.L^−1^ LiCl in acetonitrile) 10:10:1. For comparison, MALDI-TOF experiments were also carried out without mixing the analyzed sample with the matrix and cationizing salt. A small drop of the dye solution was placed onto the plate and after evaporation of the solvent was introduced for analysis in the mass spectrometer. Ions were formed by laser desorption at 337 nm N2 laser (3 ns pulse width, 100 mj per laser shots, 50 Hz maximum pulse rate). Mass spectra were collected, baseline corrected, and smoothed by Shimadzu Biotech Launchpad 2.9.9.3 software. Molar mass calculations were made by the data system using Kratos Analytical Polymer v.2.9 software. UV/VIS spectra were measured on a Shimadzu UV-1800 spectrophotometer, and the fluorescence spectra were obtained on a PerkinElmer LS45 fluorescence spectrophotometer. The width of the excitation spectrum is Δλ = λ_central_ ± 5 nm (FWHM). The fluorescence spectra were obtained on a PerkinElmer LS45 fluorescence spectrophotometer. Fresh stock solutions (1 mM) were prepared in DMSO and further diluted with TE-buffer (Tris-HCl 10 mM pH 8.0; EDTA 1 mM, pH 8.0). The absorption and emission properties of the dyes CHL and TO were investigated in TE buffer in the absence and in the presence of nucleic acids. Salmon sperm dsDNA (CAS 68938-01-2) and baker’s yeast RNA (CAS 63231-63-0) were purchased from Merck (Darmstadt, Germany) (Sigma-Aldrich, St. Louis, MO, USA) and used as received. The stock concentrations of DNA and RNA were 1 mg/mL stock. The purity of the nucleic acids was verified by their UV absorption spectra, specifically by assessing the absorbance ratio at 260/280 nm. The working concentrations of DNA and RNA solutions (10 mM Tris-HCl, 1 mM EDTA buffer, pH 8) were determined spectroscopically based on their respective molar extinction coefficients: 6600 L·mol^−1^·cm^−1^ for dsDNA [27] and 7800 L·mol^−1^·cm^−1^ for RNA [28]. All UV/VIS and fluorimetric titrations were performed by keeping the dye concentration constant while gradually adding the NA solutions of specific concentrations in the TE buffer to the dye solutions. The fluorescence spectra of the cyanine dyes were recorded in the range of 500–700 nm. After each aliquot of NA was added, the solution was allowed to equilibrate for 5 min before recording the spectra. The relative fluorescent quantum yield was defined by TO as a standard [22,23]. To avoid inner filter effects and light re-absorption, the maximal optical density at the excitation wavelength with an optical path of 1 cm was kept below 0.06. When measuring the relative quantum yields, the peak area for the maximum fluorescence intensity in the presence of the respective nucleic acid was taken into account. The relative fluorescence quantum yield of the pure dye compared to Thiazole Orange is negligibly low: 6.5 × 10^−5^. The progress of the chemical reactions is followed by thin layer chromatography (TLC) ALUGRAM^®^ SIL G/UV 254-60 Macherey-Nagel with ready-to-use plates with a thickness of the silica layer of 0.2 mm.

### 3.2. Synthesis of 3-Ethyl-2-methylbenzo[d]thiazol-3-ium Iodide (***2a***)

In an Erlenmeyer flask capped with a rubber septum under argon, 20 mL of dry acetone, 5.87 mL (5.00 g, 34.0 mmol) of 2-methylbenzothiazole, and 5.47 mL (10.50 g, 68.0 mmol) of iodoethane were added by syringe. The flask was ultrasonicated for 20 min at 25 °C. The resulting white precipitate was filtered on a Buchner funnel, washed with three portions of 30 mL of acetone, and dried in a desiccator. Yield: 9.33 g (90%). The resulting quaternary intermediate is highly hygroscopic, and therefore its chemical structure is proven by proving the structure of the final dye.

### 3.3. Synthesis of 4,7-Dichloro-1-ethylquinolin-1-ium Iodide (***2b***)

1-Ethyl-4,7-dichloroquinolinium iodide (**2b**) was synthesized in solvent-free conditions. In a high-pressure sealed tube 5.00 g (25.0 mmol) of 4,7-dichloroquinoline and 4 mL (7.80 g, 50.0 mmol) of iodoethane were placed. After saturation with argon, the reaction was capped with a Teflon cap and heated at 100 °C under intensive stirring for half an hour. The reaction mixture was cooled to room temperature, and 50 mL of dry acetone was added. The resulting precipitate was stirred with a spatula, filtered on a Buchner funnel, and washed with three portions of 50 mL dry acetone. The intermediate thus obtained was highly hygroscopic and unstable. It was stored in the dark in a vacuum desiccator. Its structure was proven by elucidating the structure of the target dye. Yield: 6.28 g (71%).

### 3.4. Synthesis of 7-Chloro-1-ethyl-4-((3-ethylbenzo[d]thiazol-2(3H)-ylidene)methyl)quinolin-1-ium Iodide (CHL)

#### 3.4.1. Procedure A

An amount of 0.50 g (1.6 mmol) 3-ethyl-2-methyl-benzo[d]thiazolium iodide and 0.69 g (1.9 mmol) 4,7-dichloro-1-ethylquinolinium iodide, 0.37 g (3.5 mmol) dry sodium carbonate, and one drop DIPEA were ground in a mortar for 15 min. After adding 3 mL ethanol and 3 mL distilled water, grinding continued for another 15 min. The mixture was transferred to a 100 mL Becher receptable and was filled with 50 mL distilled water. The red precipitate was filtered, washed with water and ethanol, and dried in a desiccator. Yield: 0.48 g (65%). The analytical sample was obtained by fractional recrystallization from ethanol. Mp > 250 °C. ^1^H-NMR (500 MHz, DMSO-*d*_6_, δ (ppm)): 8.82 d (1H, CH, J = 9 Hz), 8.59 d (1H, CH, J = 7 Hz), 8.24 s (1H, CH), 8.08 d (1H, CH, J = 8 Hz), 7.83 d (1H, CH, J = 8 Hz), 7.76 d (1H, CH, J = 9 Hz), 7.64 dd (1H, CH, J_1_ = J_2_ = 8 Hz), 7.46 dd (1H, CH, J_1_ = J_2_ = 8 Hz), 7.37 d (1H, CH, J = 7 Hz), 6.92 s (1H, CH), 4.70 q (2H, J = 7 Hz, N^+^CH_2_), 4.61 q (2H, J = 7 Hz, N^+^CH_2_), 1.45 t (3H, CH_3_, J = 7 Hz), 1.38 t (3H, CH_3_, J = 7 Hz). ^13^C-NMR (125 MHz, DMSO-d_6_, δ (ppm)): 160.26 (C), 148.74 (C), 144.76, 139.99 (C), 138.81 (C), 138.30 (C), 128.90, 128.53, 127.39, 125.31, 124.73 (C), 123.57, 123.43 (C), 117.75, 113.53, 108.78, 88.28, 66.82, 49.86, 15.10, 12.90. ^13^C-DEPT-NMR (135 MHz, DMSO-*d*_6_, δ (ppm)): 144.77 (CH), 128.91 (CH), 128.54 (CH), 127.39 (CH), 125.31 (CH), 123.58 (CH), 117.76 (CH), 113.54 (CH), 108.78 (CH), 88.28 (CH), 49.87 (N^+^CH_2_), 41.73 (N^+^CH_2_), 15.11 (CH_3_), 12.89 (CH_3_). MALDI-TOF (*m*/*z*) Calculated Chemical Formula: C_21_H_20_ClN_2_S^+^; Calculated Exact Mass = 367.91; Found Mass [*m*/*z*]^+^ = 367.4195 (100%).

#### 3.4.2. Procedure B

By the classical conditions 0.50 g (1.6 mmol) 3-ethyl-2-methyl-benzo[d]thiazolium iodide and 0.69 g (1.9 mmol) 4,7-dichloro-1-ethylquinolinium iodide, 0.37 g (3.5 mmol) was ground in a mortar. The mixture was transferred to a 50 mL round-bottom flask equipped with an electromagnetic stirrer and a rubber septum. The flask was flushed with argon, and a mixture of 7 mL ethanol and 7 mL dichloromethane was added by syringe. After adding by syringe 0.61 mL (0.45 g, 3.5 mmol) DIPEA, the reaction was stirred vigorously at room temperature for 1 h. The obtained red precipitate was filtered, washed with water and ethanol, and dried in a desiccator. Yield: 0.62 g (89%). The precipitate was compared by TLC in eluent 4 mL DCM–0.3 mL ethanol. The product from procedure A was used as a reference.

### 3.5. Computational Details

The geometry optimization and photophysical properties of the newly synthesized dye CHL, its aggregates, TO, and ID1 were computed using the G16 software package [29]. Ground-state energy minimum structures were optimized at the DFT level of theory, employing an M062X functional [17] in combination with a 6-31G(d,p) basis set [18]. For iodide counterions, the SDD basis set and corresponding effective core potential were applied [19]. To simulate experimental conditions (TE buffer), all computations were performed in an aqueous medium, incorporating solvent effects at every step via the polarizable continuum model (PCM) formalism [30,31]. To ensure that each optimized structure corresponded to a true minimum on the potential energy surface, harmonic vibrational frequency analysis was conducted at the same level of theory. No imaginary frequencies were observed. Theoretical absorption spectra in water were also estimated using TDDFT. The absorption maxima were determined by evaluating the lowest-energy vertical excitation transitions. For these calculations, the HFS functional [32,33] and the 6-31+G(d,p) basis set [18] were employed.

## 4. Conclusions

An environmentally friendly synthetic procedure was developed for the preparation of monomethine cyanine dyes. The photophysical properties of the newly synthesized monomethine cyanine dye, CHL, were investigated using both experimental and theoretical approaches. The binding affinity and interaction mode of the dye with nucleic acids were evaluated using fluorescence titration, providing further insight into their nucleic acid recognition capabilities. CHL interacts with both dsDNA and RNA, forming fluorescent complexes that show a substantial increase in fluorescence intensity upon binding. CHL demonstrates a marked sensitivity toward RNA, with up to a 125-fold enhancement in fluorescence intensity upon complex formation. The obtained results unequivocally prove that the dye CHL is suitable for all types of nucleic acid analyses and visualization.

## Figures and Tables

**Table 1:** Experimental and PCM/HFS/6-31+G(d,p) calculated absorption maxima in nm and molar absorptivity ε (in L·mol^−1^·cm^−1^) for the monomeric dyes and the most stable π-stacked dimers of TO and CHL.

Dye	Monomer TE Buffer		Monomer Ethanol	Dimer TE Buffer	
	$\lambda_{exp}$ [nm]	$\lambda_{calc}$ [nm]	$\lambda_{exp}$ [nm]	$\lambda_{exp}$ [nm]	$\lambda_{calc}$ [nm]
CHL	509 (48,694)	505	511 (85,834)	482	474
TO	500 (93,710)	496	503 (196,691)	476	471

**Table 2:** Fluorescent properties of the dyes and their complexes with nucleic acids. I_0_ values refer to the fluorescence of the dyes neat in TE buffer in concentrations given in Figure 5. The fluorescence of the complexes (I_max_) refers to the highest NA concentration, as presented in Figure 5.

Dye	NA	$\lambda_{ex}$ (nm)	$\lambda_{em}^{dye}$ (nm)	$\lambda_{em}^{complex}$ (nm)	I_max_/I_0_	K_b_ × 10^5^	Φ_f_
CHL ^a^	DNA	489	547	538	76	3.74	0.33
	RNA	491	545	541	125	1.65	0.14
TO ^b^	DNA	476	537	533	216	3.13	0.25
	RNA	476	534	532	93	4.25	0.40

^a^ The relative fluorescent quantum yields were determined in TE buffer pH = 7.4 with Thiazole Orange using literature conditions [22]. ^b^ Standard of the relative fluorescence quantum yield was performed with sodium fluorescein (Φf = 0.85, in MeOH/TE buffer) [23]. The relative fluorescence quantum yield of the pure dye CHL compared to Thiazole Orange is negligibly low 6.5 × 10^−5^.

## Data Availability

The original contributions presented in this study are included in the article/Supplementary Materials. Further inquiries can be directed to the corresponding authors.

## Supplementary Materials

The following supporting information can be downloaded at https://www.mdpi.com/article/10.3390/molecules30193966/s1, Schemes S1–S3. ^1^H-NMR spectra of the dye CHL in deuterated DMSO. Scheme S4. ^13^C-NMR spectra in the range of 0–170 ppm of the dye CHL in deuterated DMSO. Scheme S5. ^1^H-NMR spectra in the range of 0–72 ppm of the dye CHL in deuterated DMSO. Scheme S6. ^13^C-NMR spectra in the range 90–164 ppm of dye CHL in deuterated DMSO. Schemes S7–S9. ^13^C-DEPT-135 NMR spectra of the dye CHL in deuterated DMSO. Scheme S10. HSQC NMR of the dye CHL in DMSO-*d*_6_. Scheme S11. MALDI-TOF mass spectra of the dye CHL. Figure S1. PCM/M062X/6-31G(d,p) optimized geometry of the different H-dimers of the dye CHL. Figure S2. PCM/M062X/6-31G(d,p) optimized geometry of the different J-dimers of the dye CHL. Table S1. The relative to the most stable dimer free energies for each of the eight studied dimers of the dye CHL. PCM/M062X/6-31G(d,p) optimized Cartesian Coordinates for the most stable H- and J-dimers of CHL. Reference [20] is cited in the Supplementary Materials.
