# Supplementary material for: Mechanochemical Approach to a Monocationic Asymmetric Monomethine Cyanine Dye for Nucleic Acid Analysis and Visualization
Source: Molecules. 2025 Oct 2;30(19):3966. doi: 10.3390/molecules30193966 (PMC12526355; doi:10.3390/molecules30193966)
Supplement: Supplementary file 1 [file molecules-30-03966-s001.zip › molecules-3860979-supplementary.pdf]

Electronic supporting information

Mechanochemical approach to a monocationic asymmetric monomethine cyanine dye for nucleic acid analysis and visualization

Diana Cheshmedzhieva[1,*], Nadezhda Bozova[1], Sonia Ilieva[1], Christo Novakov[2], Aleksey Vasilev[1,2,*]

[1] Faculty of Chemistry and Pharmacy, Sofia University "St. Kliment Ohridski", 1 J. Bourchier Ave., 1164 Sofia, Bulgaria; e-mail: ohtdv@chem.uni-sofia.bg; silieva@chem.uni-sofia.bg; nadegdabozova@abv.bg; ohtavv@chem.uni-sofia.bg

[2] Institute of Polymers, Bulgarian Academy of Sciences, Akad. G. Bonchev St., bl 103A, 1113 Sofia, Bulgaria.; e-mail: hnovakov@polymer.bas.bg; a_vassilev@polymer.bas.bg

*Correspondence: a_vassilev@polymer.bas.bg (A.V.), ohtdv@chem.uni-sofia.bg (D.C.)

Contents:

Scheme S1. Full range ^{1}H-NMR spectra of dye CHL in deuterated DMSO. **S2**

Scheme S2. ^{1}H-NMR spectra in the range 0-5 ppm of dye CHL in deuterated DMSO. **S2**

Scheme S3. ^{1}H-NMR spectra in the range 6-9.2 ppm of dye CHL in deuterated DMSO. **S3**

Scheme S4. ^{13}C-NMR spectra in the range 0-170 ppm of dye CHL in deuterated DMSO. **S3**

Scheme S5. ^{1}H-NMR spectra in the range 0-72 ppm of dye CHL in deuterated DMSO. **S4**

Scheme S6. ^{13}C-NMR spectra in the range 90-164 ppm of dye CHL in deuterated DMSO. **S4**

Scheme S7. ^{13}C-DEPT-135 NMR spectra in the range 0-164 ppm of dye CHL in deuterated DMSO. **S5**

Scheme S8. ^{13}C-DEPT-135 NMR spectra in the range 0-164 ppm of dye CHL in deuterated DMSO. **S5**

Scheme S9. ^{13}C-DEPT-135 NMR spectra in the range 0-90 ppm of dye CHL in deuterated DMSO. **S6**

Scheme 10. HSQC NMR of dye CHL in DMSO-d_6. **S6**

Scheme S11. MALDI-TOF mass spectra of dye CHL. **S7**

Figure S1. PCM/M062X/6-31G(d,p) optimized geometry of the different H- dimer of the dye CHL in a water medium: left—side view, right—top view. **S9**

Figure S2. PCM/M062X/6-31G(d,p) optimized geometry of the different J- dimer of the dye CHL in a water medium: left—side view, right—top view. **S10**

Table S1. The relative to the most stable dimer free energies for each of the eight studied dimers of dye CHL. The structure of the dimers were optimized at PCM/M062X/6-31G(d,p) level of theory. **S11**

Cartesian coordinates, electronic and free energies and the number of imaginary frequencies (all in atomic units) for the PCM/M062X/6-31G(d,p) optimized geometry of the most stable H- and J-dimers of dye CHL. **S11**

References **S18**

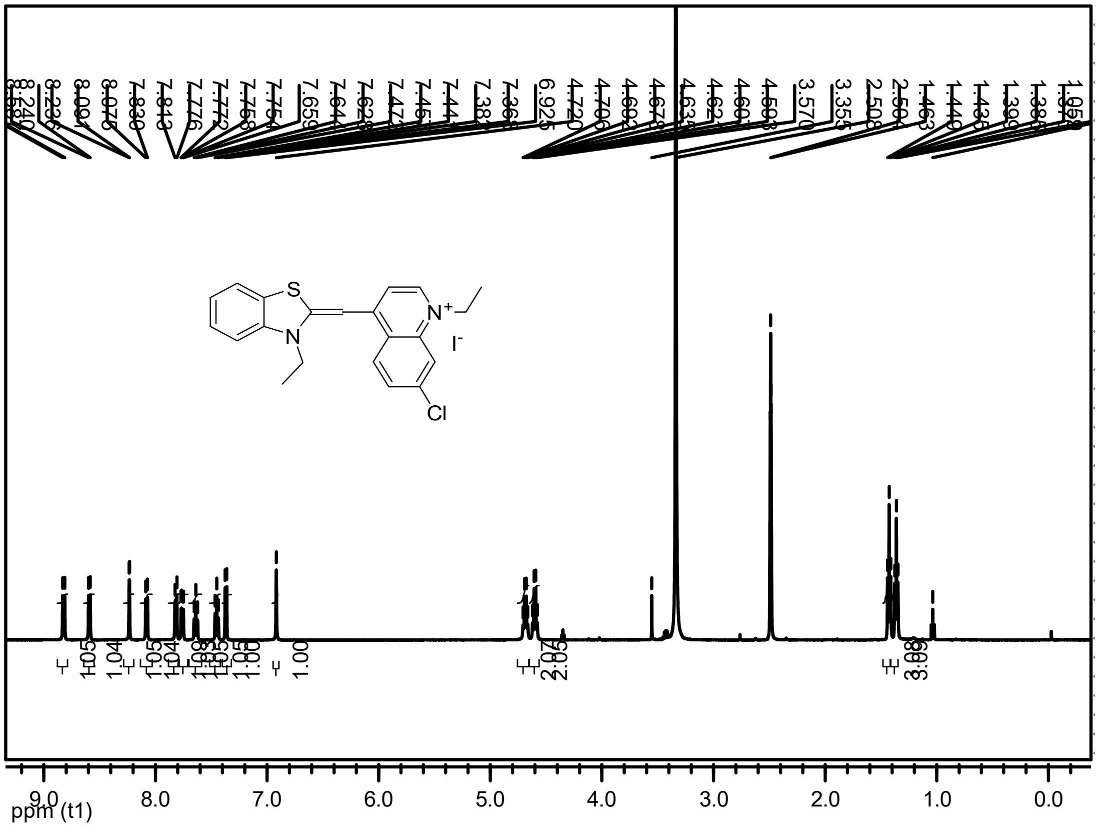

Scheme S1. Full range ^{1}H-NMR spectra of dye CHL in deuterated DMSO.

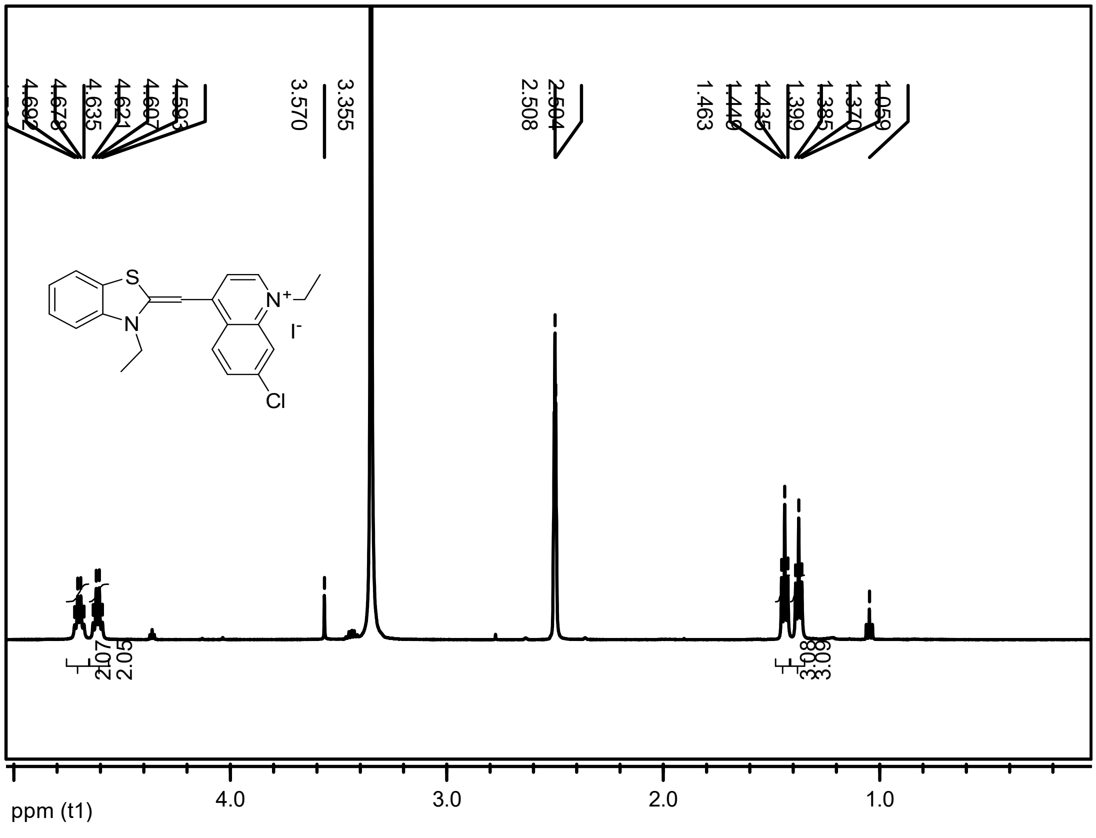

Scheme S2. ^{1}H-NMR spectra in the range 0-5 ppm of dye CHL in deuterated DMSO.

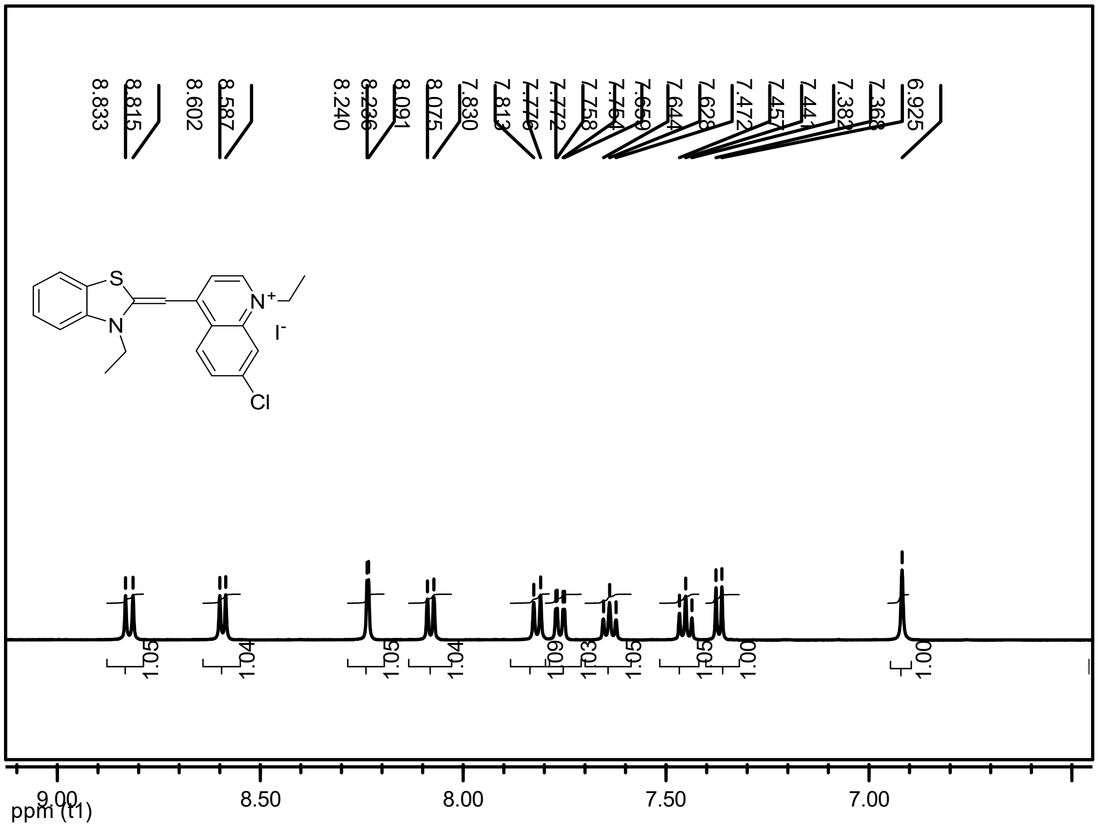

Scheme S3. ^{1}H-NMR spectra in the range 6-9.2 ppm of dye CHL in deuterated DMSO.

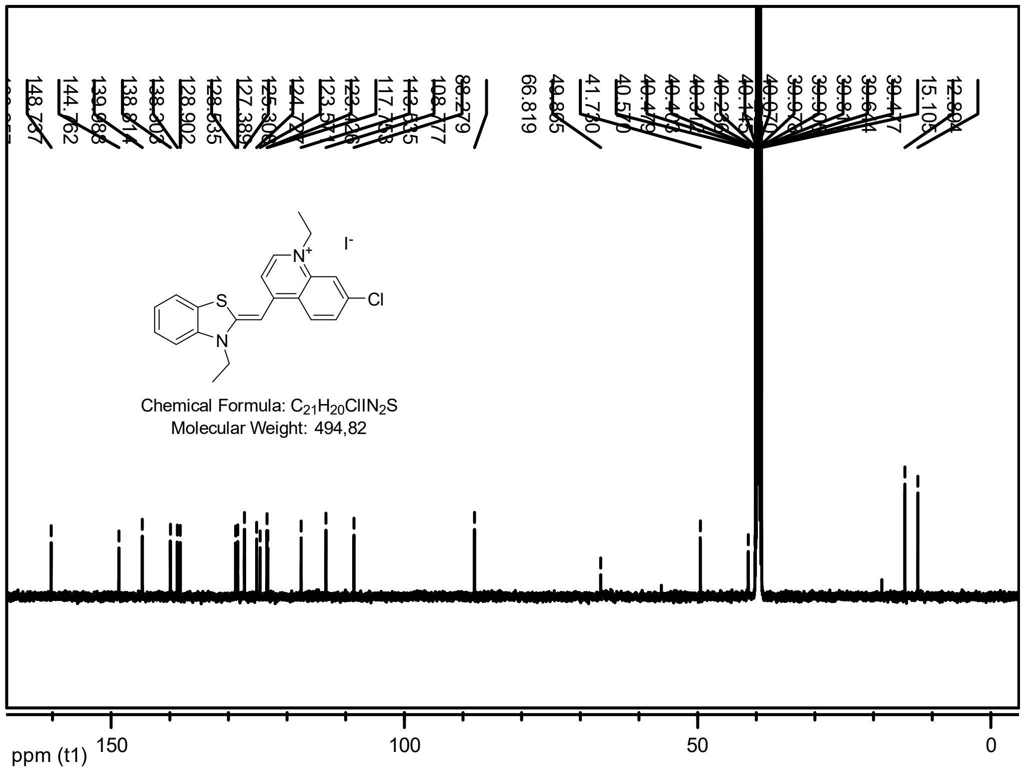

Scheme S4. ^{13}C-NMR spectra in the range 0-170 ppm of dye CHL in deuterated DMSO.

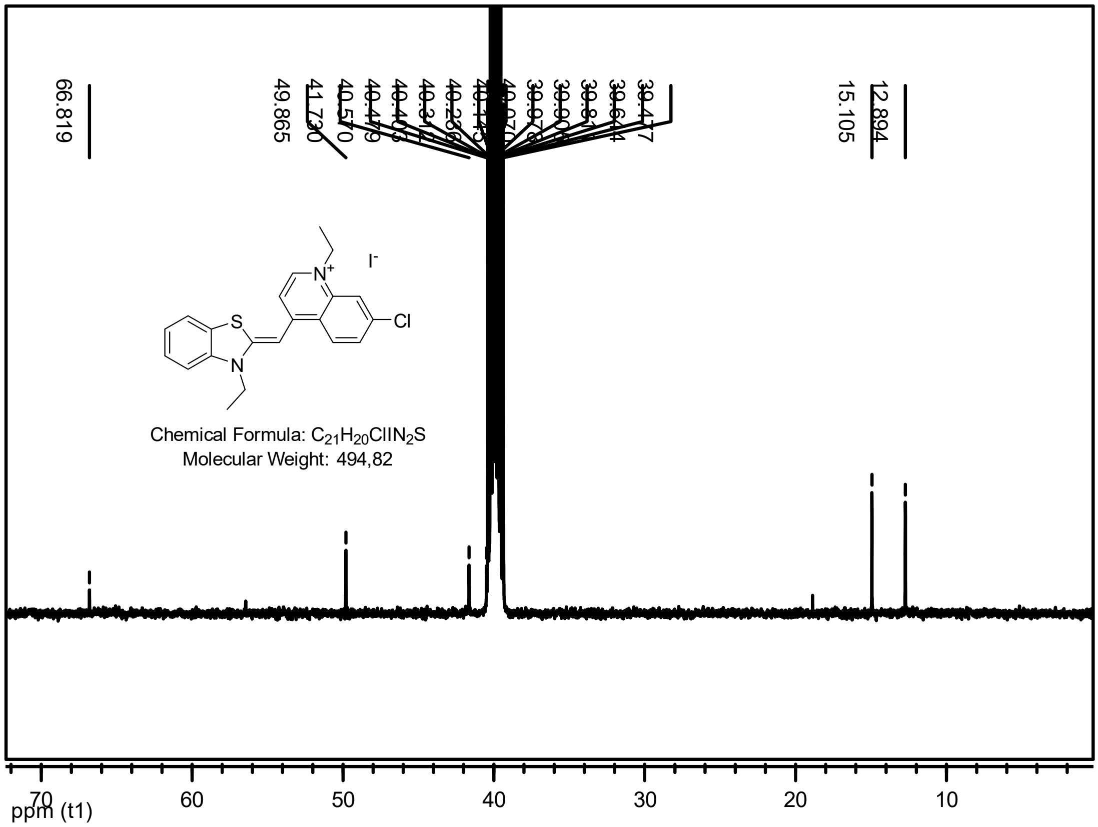

Scheme S5. ^{1}H-NMR spectra in the range 0-72 ppm of dye CHL in deuterated DMSO.

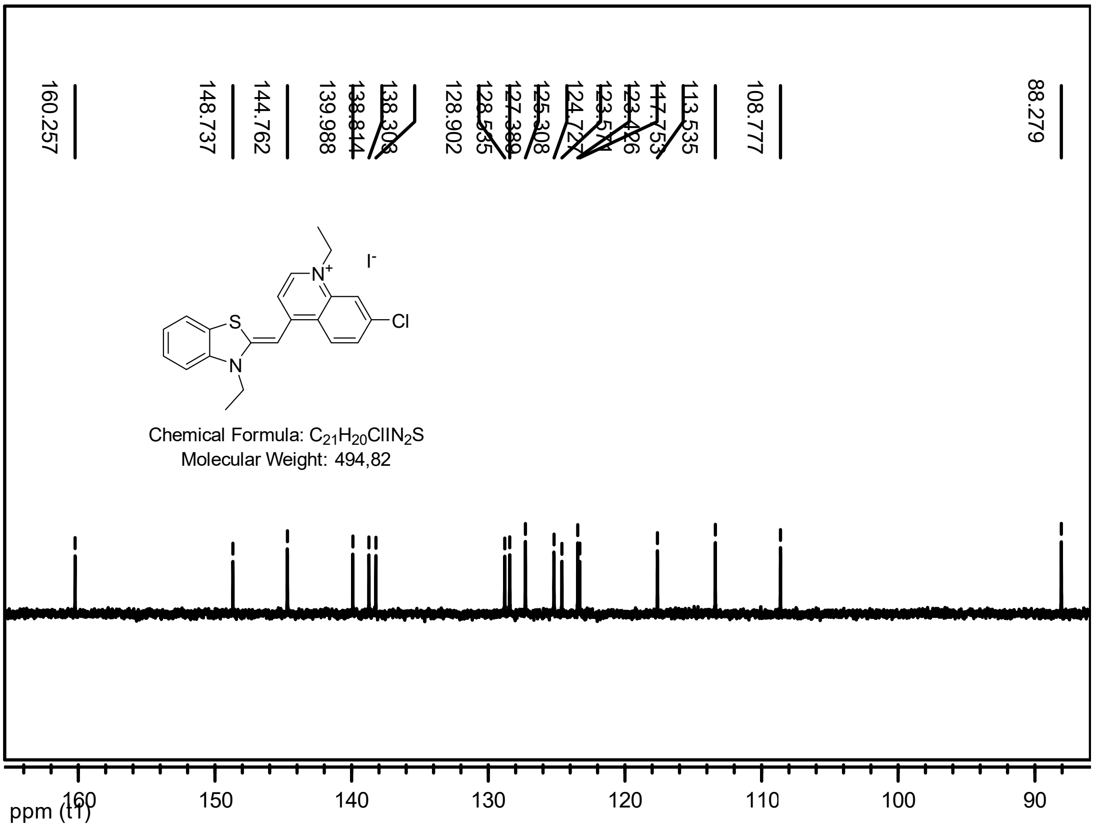

Scheme S6. ^{13}C-NMR spectra in the range 90-164 ppm of dye CHL in deuterated DMSO.

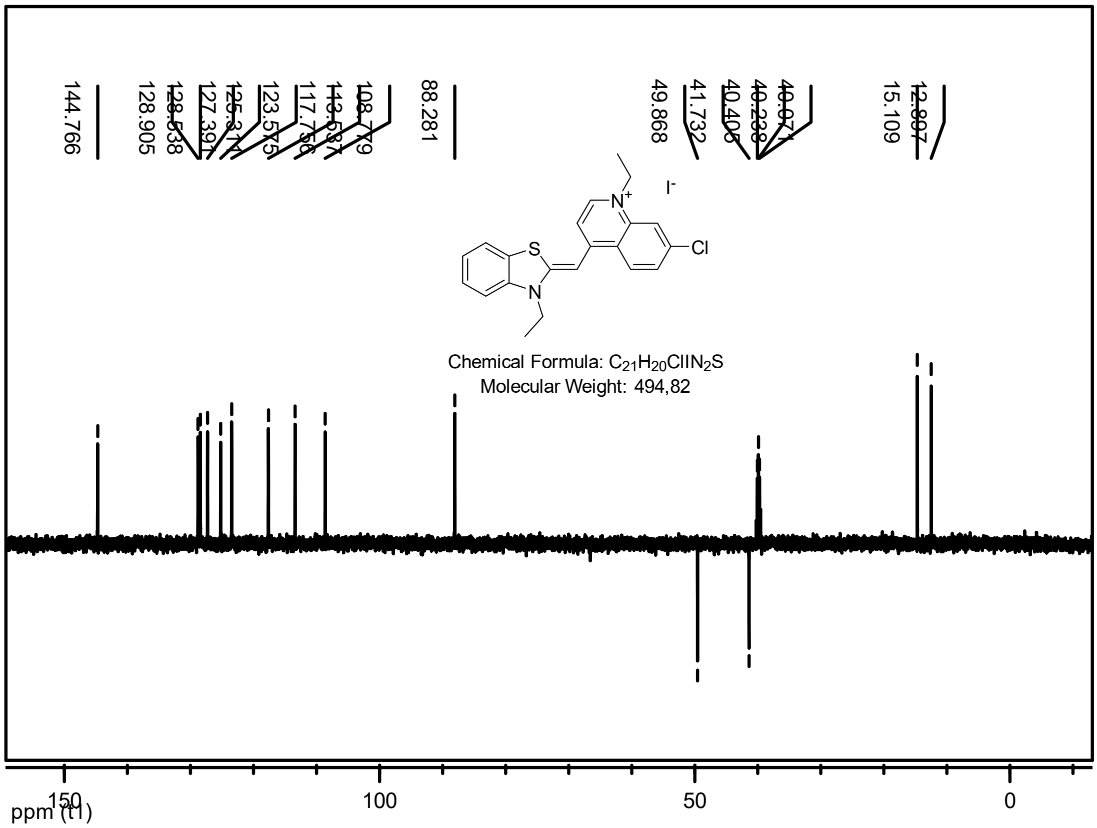

Scheme S7. ^{13}C-DEPT-135 NMR spectra in the range 0-164 ppm of dye CHL in deuterated DMSO.

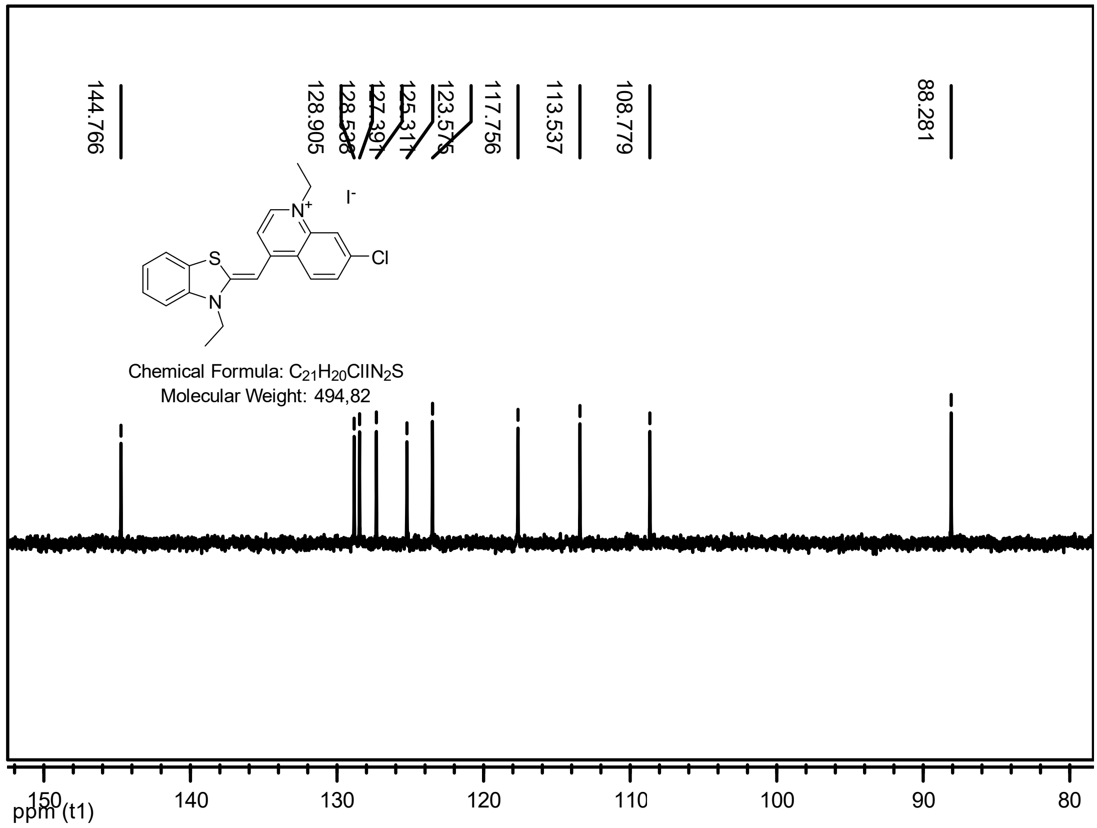

Scheme S8. ^{13}C-DEPT-135 NMR spectra in the range 0-164 ppm of dye CHL in deuterated DMSO.

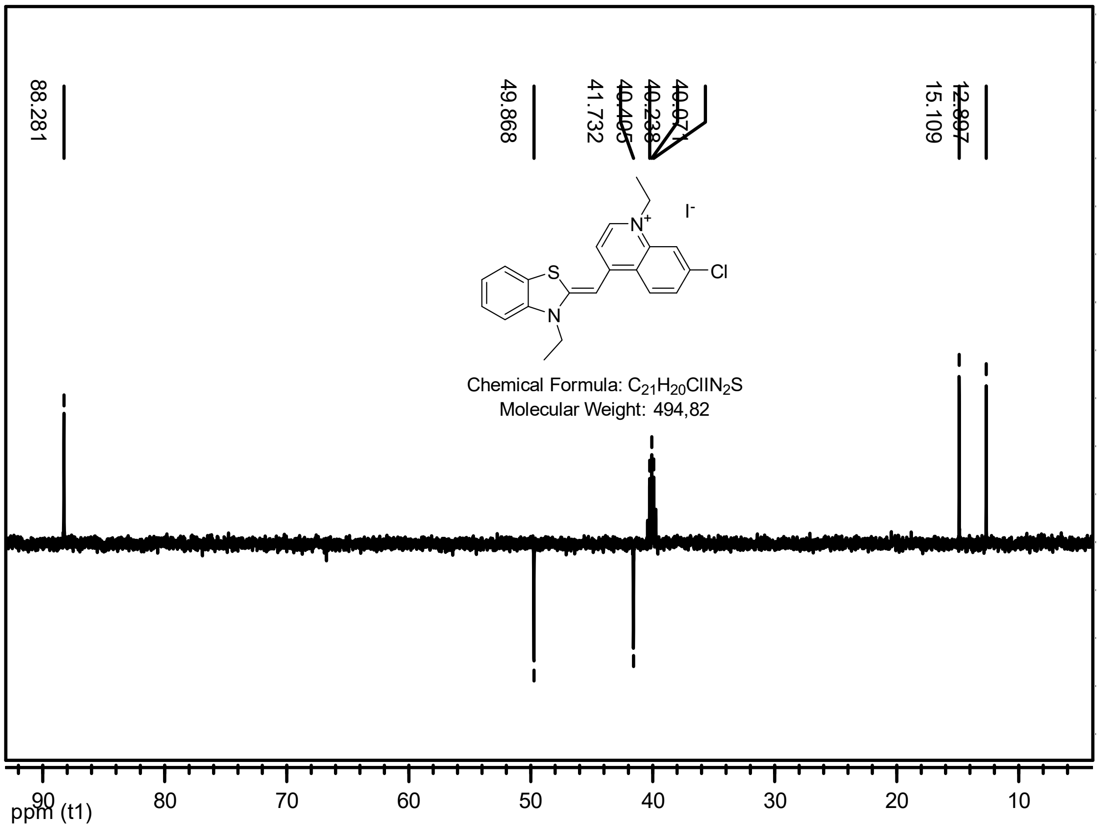

Scheme S9. ^{13}C-DEPT-135 NMR spectra in the range 0-90 ppm of dye CHL in deuterated DMSO.

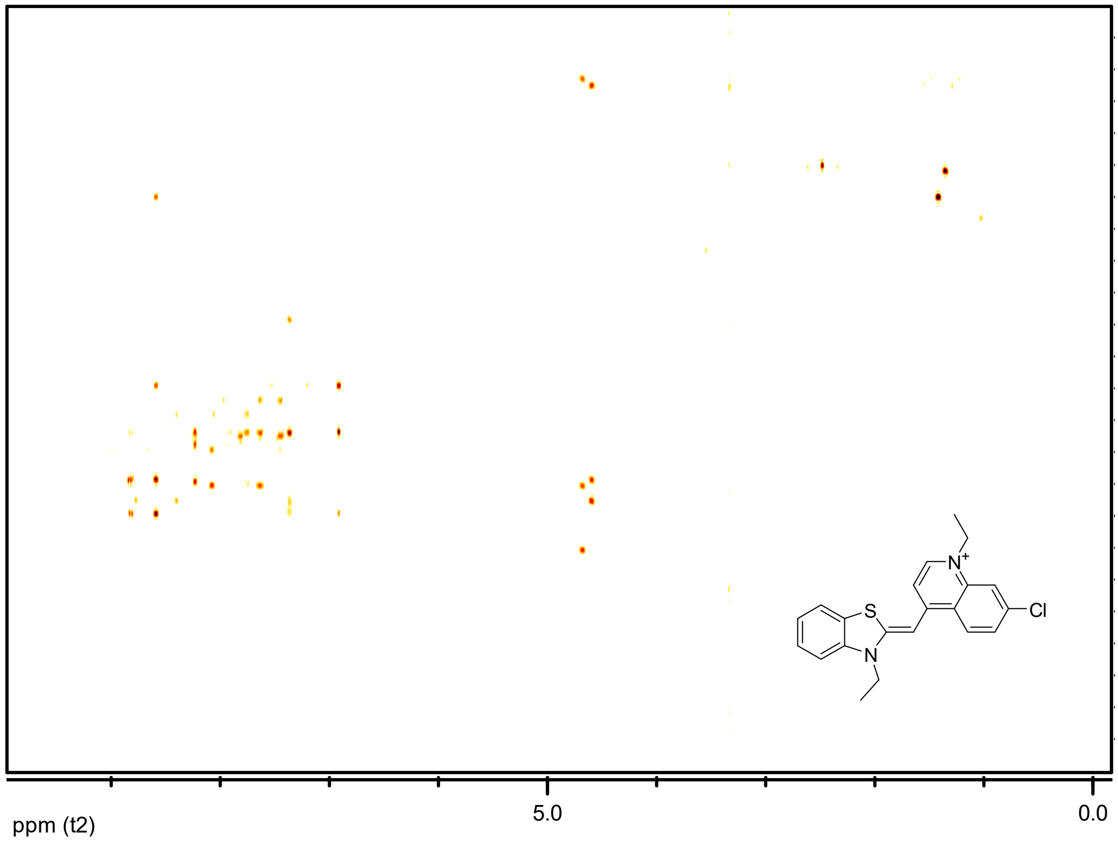

Scheme 10. HSQC NMR of dye CHL in DMSO-*d*$_6$.

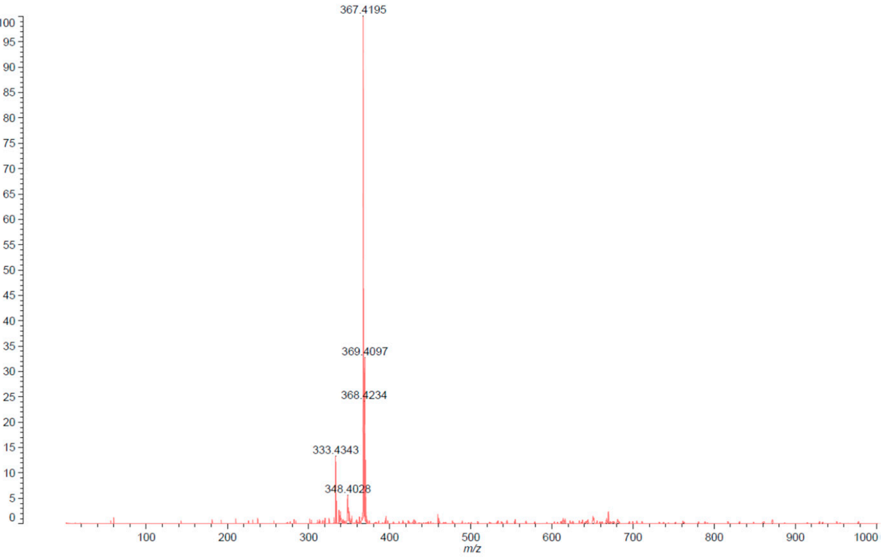
367.4195
369.4097
368.4234
333.4343
348.4028
m/z

a)

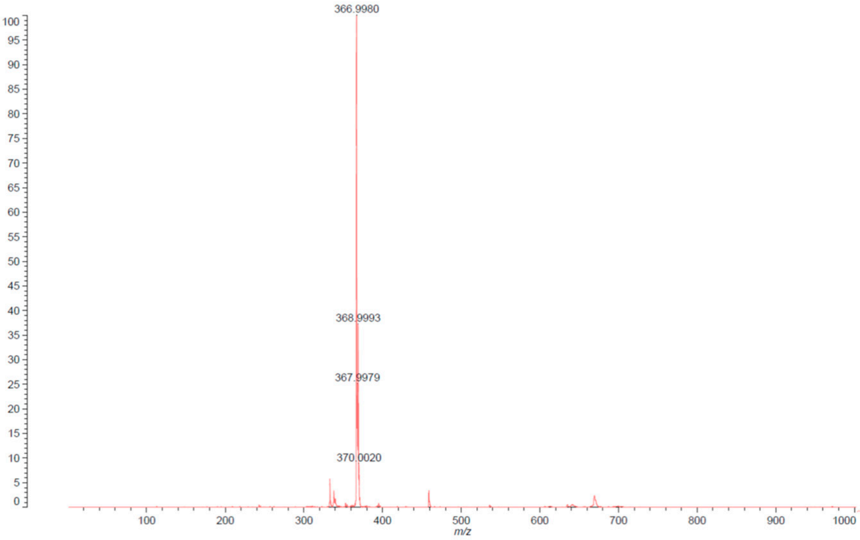
366.9980
368.9993
367.9979
370.0020
m/z

b)

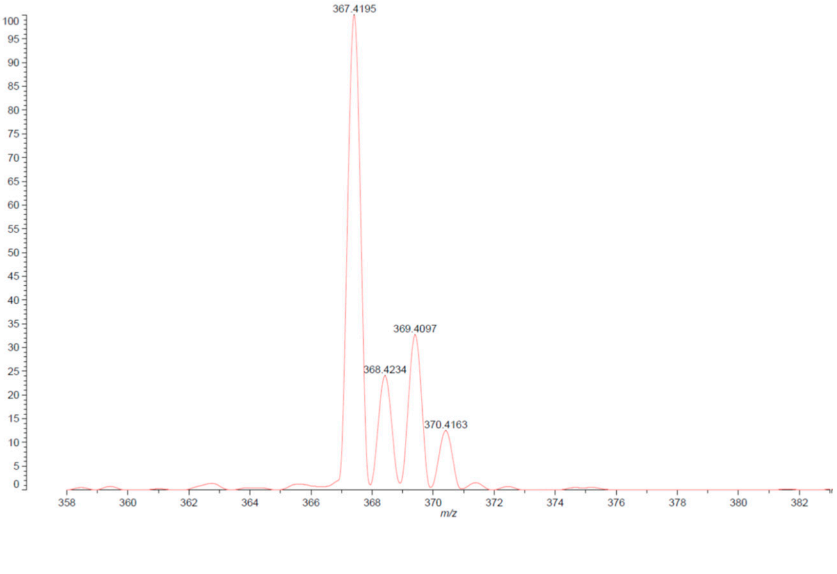
367.4195
369.4097
368.4234
370.4163
m/z

c)

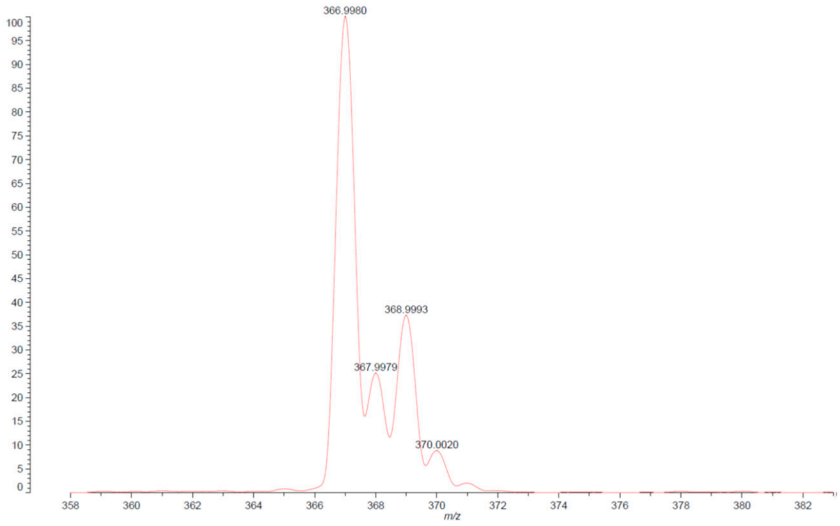

d)

Scheme S11. MALDI-TOF mass spectra of dye CHL: a) full mass spectrum taken in reflectron operating mode; b) full mass spectrum taken in linear operating mode; c) detailed view of the main molecular ion peak taken in reflectron operating mode; d) detailed view of the main molecular ion peak taken in linear operating mode

Top view | Side view

H1 dimer

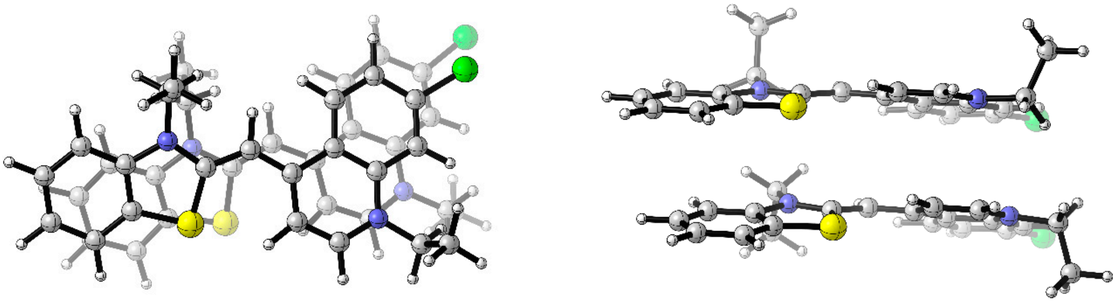

H2 dimer most stable

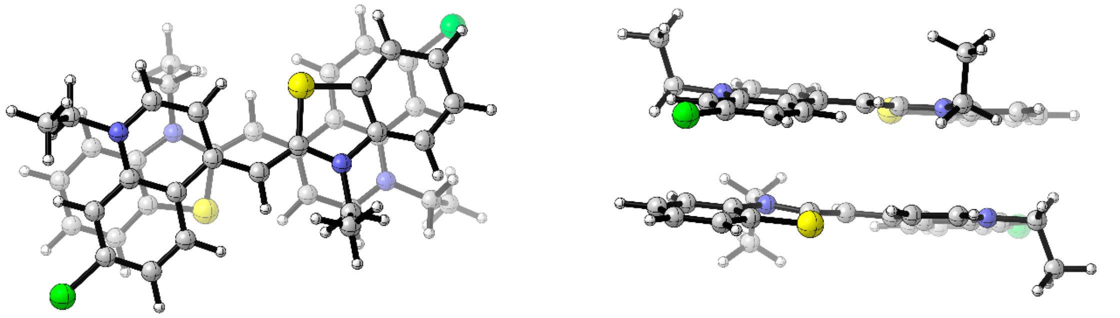

H3 dimer

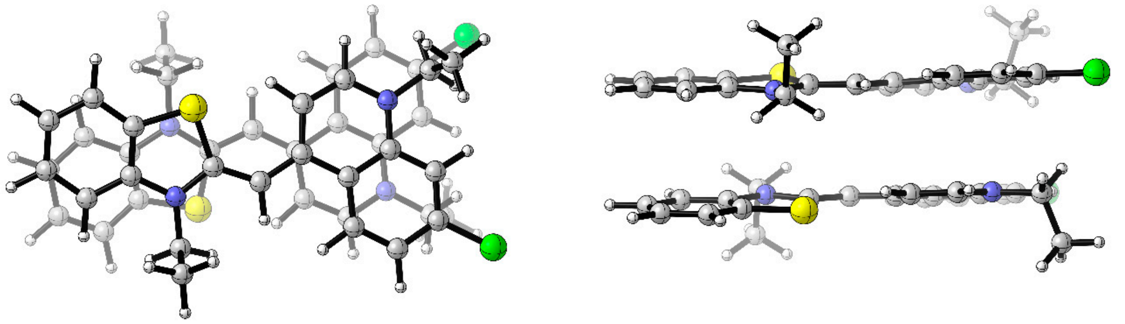

H4 dimer

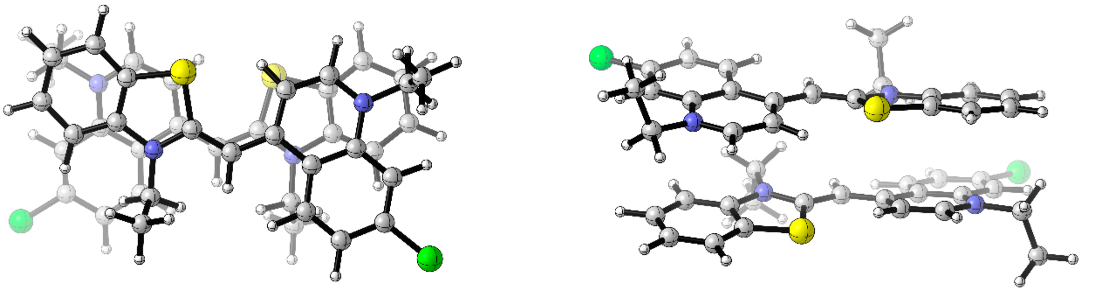

Figure S1. PCM/M062X/6-31G(d,p) optimized geometry of the different H- dimer of the dye CHL in a water medium: left—side view, right—top view. Color scheme: O—red, N—blue, S—yellow, C—gray, H—light gray [1].

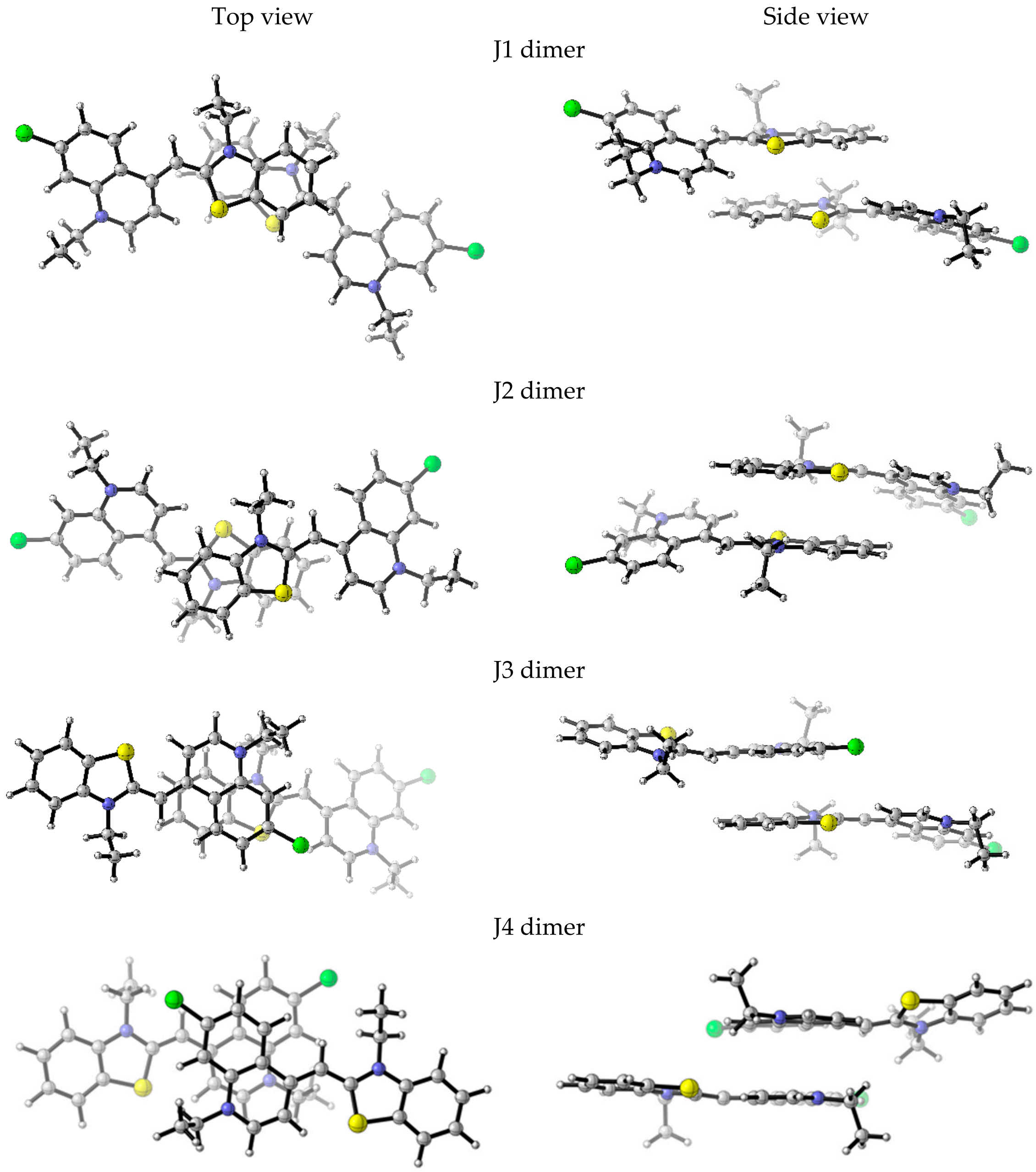

Figure S2. PCM/M062X/6-31G(d,p) optimized geometry of the different J- dimer of the dye CHL in a water medium: left—side view, right—top view. Color scheme: O—red, N—blue, S—yellow, C—gray, H—light gray [1].

Table S1. The relative to the most stable dimer free energies for each of the eight studied dimers of dye CHL. The structure of the dimers were optimized at PCM/M062X/6-31G(d,p) level of theory.

Dimer	ΔΔ G [kcal/mol]
H1	2.3
H2	0.0
H3	0.8
H4	0.2
J1	5.9
J2	6.5
J3	7.5
J4	4.7

PCM/M062X/6-31G(d,p) optimized Cartesian coordinates, electronic and free energies and the number of imaginary frequencies (all in atomic units) for the most stable H- and J-dimers of dye CHL.

H1

2 1
C,0,-6.1619522638,-0.8415245865,-2.5626183996
C,0,-5.6526448854,0.1703566579,-1.7592485638
C,0,-4.2724107026,0.3844081727,-1.7746307451
C,0,-3.4433245092,-0.3855183843,-2.5929928388
C,0,-3.9530660182,-1.4088294129,-3.3860916505
C,0,-5.3254935295,-1.627785105,-3.3632837705
N,0,-3.5550772123,1.320532461,-1.0265238511
C,0,-2.2200219467,1.3647316892,-1.2784552857
S,0,-1.7786256442,0.1317218799,-2.441764308
C,0,-1.3777428381,2.3374969116,-0.7377045692
C,0,0.0204306303,2.4334884296,-0.8510163698
C,0,0.7098866486,3.6653772097,-0.4827680484
C,0,2.1240858756,3.7510484253,-0.5667174052
N,0,2.8570113359,2.658305027,-1.0090051646
C,0,2.2159144627,1.526292862,-1.3529634924
C,0,0.8556560429,1.3765571192,-1.2832280098
C,0,0.0135184217,4.8192018535,-0.0574960364
C,0,0.6585247361,5.9803872242,0.2958509872
C,0,2.0575140214,6.0197878181,0.2244469711
C,0,2.7916289229,4.9373045687,-0.2012569747
H,0,-7.2301830021,-1.0271164089,-2.5591716576
H,0,-6.3149242905,0.7711580055,-1.1467198252
H,0,-3.2981773348,-2.0079175609,-4.0083995231
H,0,-5.7501510641,-2.4162860664,-3.9741142902
H,0,-1.8872249318,3.1248240218,-0.2042477769
H,0,2.8482031999,0.7103574644,-1.6859006131
H,0,0.4664814188,0.3940546522,-1.5067326361
H,0,-1.0674792543,4.8128936227,-0.0109260412
H,0,0.1026944981,6.8522503829,0.6182005505
H,0,3.8688543294,5.0169292341,-0.2456859497
C,0,4.3145636466,2.7475660498,-1.2405052537
H,0,4.6839666699,1.7220232619,-1.2825266757
C,0,-4.2329028905,2.2518594347,-0.1198993474
H,0,-3.5524050314,2.4571399081,0.7092642248
H,0,-5.0889470242,1.7181967638,0.299107166
H,0,4.7770752481,3.2176408692,-0.371038822
C,0,-7.2075661858,-2.2740269589,0.7326929331
C,0,-6.5287863148,-1.297748469,1.453984715
C,0,-5.141118998,-1.2316878441,1.3207242947
C,0,-4.4702472284,-2.1365932127,0.4937344879
C,0,-5.1470934246,-3.1143154953,-0.2239164817
C,0,-6.5306835056,-3.1730282829,-0.0966915939
N,0,-4.270359738,-0.3246469956,1.933339029
C,0,-2.9520206069,-0.4991976788,1.6365608828
S,0,-2.7491836942,-1.8315192588,0.5179558773
C,0,-1.9454588488,0.3339485776,2.118712427
C,0,-0.5556009546,0.2502840943,1.8914306085
C,0,0.3089042785,1.3735379459,2.2351965735
C,0,1.7169875801,1.2716206035,2.083621239
N,0,2.270285146,0.1073336531,1.5731384314
C,0,1.4697445591,-0.9166795564,1.2388882402
C,0,0.1039205772,-0.8820785955,1.3665506477
C,0,-0.2068563364,2.6073402427,2.6919936616
C,0,0.6052458355,3.6641278429,3.0290953653
C,0,1.9939354496,3.5124672528,2.9115778714
C,0,2.5559077222,2.3471999281,2.4422939621
H,0,-8.2861397466,-2.3371398844,0.8236571104
H,0,-7.0723090792,-0.6201740575,2.1011159103
H,0,-4.6102665492,-3.8012451368,-0.8683459302
H,0,-7.0868502625,-3.9223000392,-0.6484917053
H,0,-2.2875981058,1.1390178944,2.7505326041
H,0,1.9705605595,-1.8033414495,0.8682033946
H,0,-0.4176029575,-1.7950863563,1.1252217617
H,0,-1.2766985405,2.7535110676,2.7648489426
H,0,0.1863239043,4.6032530486,3.369879965
H,0,3.6326297604,2.2749305361,2.3711131343

C,0,3.7320301983,-0.0675361009,1.4537372591
H,0,4.1512992072,0.8466531948,1.0293657151
H,0,3.8907001997,-0.8586326434,0.7201733986
C,0,-4.7472543391,0.6293357704,2.942956631
H,0,-4.1796051114,1.5554622341,2.8422294709
H,0,-5.7784448266,0.8766089284,2.6887451158
C,0,4.3733257734,-0.428904387,2.786617444
H,0,3.9663685322,-1.3725515553,3.1559642076
H,0,5.4497952283,-0.5436403792,2.6481268443
H,0,4.2018121295,0.3418003316,3.5409603412
C,0,4.6388497251,3.4817718482,-2.534911166
H,0,5.7219055724,3.5182201195,-2.6653832762
H,0,4.2023746393,2.9551408137,-3.3862602524
H,0,4.2577919371,4.505111599,-2.5264570721
C,0,-4.6410054155,0.0528299473,4.3483790155
H,0,-5.0100294575,0.7804840617,5.0733055387
H,0,-5.2380479407,-0.8582360759,4.432208114
H,0,-3.6024136642,-0.185292422,4.5903514596
C,0,-4.6708871535,3.5247186348,-0.832647959
H,0,-5.3686456767,3.2884562946,-1.6394724555
H,0,-5.1689851085,4.189199437,-0.1245923839
H,0,-3.8136639784,4.0493404893,-1.2614672066
Cl,0,3.0284673203,4.8303107271,3.3676356933
Cl,0,2.8849001488,7.4768642308,0.6797468395
E_{el} = 3559.2539673
G =-3558.595247
Nimag =0

H2

2 1
C,0,-5.8168327723,-1.8892361942,-2.3624591133
C,0,-5.4161634077,-0.6676100393,-1.8304703082
C,0,-4.0486906708,-0.3883767004,-1.7864723087
C,0,-3.1253591348,-1.3118317184,-2.284630881
C,0,-3.5263026263,-2.5315665571,-2.8193970849
C,0,-4.8874454489,-2.8133627049,-2.8518623501
N,0,-3.4329839005,0.7547423947,-1.2731891009
C,0,-2.0770101086,0.7835117102,-1.3958439332
S,0,-1.4985739057,-0.6888510526,-2.1414025454
C,0,-1.3073978784,1.8796632576,-1.0102928753
C,0,0.0938268795,2.0044294658,-0.9708066531
C,0,0.6985274554,3.2956381888,-0.660659417
C,0,2.1030993828,3.4124879753,-0.4962832944
N,0,2.9119364605,2.3003982438,-0.673994819
C,0,2.3568432926,1.1255726018,-1.0178547051
C,0,1.0057956958,0.9395801915,-1.1604420263
C,0,-0.0728573186,4.4709580777,-0.5027631768
C,0,0.4881683209,5.6775333359,-0.1625381508
C,0,1.8762127237,5.7455869654,0.0264491171
C,0,2.6845290521,4.6480658795,-0.1438884455
H,0,-6.8756526876,-2.1203702281,-2.4035635152
H,0,-6.1546382027,0.0445639625,-1.480047788
H,0,-2.7961917167,-3.2399253775,-3.1956660756
H,0,-5.2281896407,-3.7575673395,-3.2608306226
H,0,-1.8819815064,2.7340587055,-0.6916935043
H,0,3.0482884536,0.3025460926,-1.1597570686
H,0,0.6856604813,-0.0705995322,-1.3639730389
H,0,-1.1421438985,4.4449100742,-0.6649547348
H,0,-0.122968275,6.563966228,-0.0446785163
H,0,3.7506648361,4.7505979281,0.0020232001
C,0,4.384569389,2.3968545969,-0.5933047383
H,0,4.7531582927,1.3818820366,-0.4399279314
C,0,-4.2160255354,1.9047640048,-0.8033226281
H,0,-3.6793638272,2.360464408,0.0306744685
H,0,-5.1452233936,1.5109201223,-0.389295042
H,0,4.6393060546,2.9655073989,0.3027943191
C,0,3.5147381228,4.0329945249,3.1534425578
C,0,3.2351094879,2.7160863409,2.8025751761
C,0,1.8997836023,2.3605774041,2.5982691861
C,0,0.8861670222,3.308857483,2.7606796246
C,0,1.1659769665,4.6250422966,3.1126321658
C,0,2.4963810686,4.9794174612,3.3083938449
N,0,1.4016559054,1.1081895442,2.2348142769
C,0,0.0438547352,1.0230033037,2.1597123496
S,0,-0.6811634861,2.5837014571,2.4845566955
C,0,-0.6197313223,-0.1526169885,1.8132588776
C,0,-2.0051334078,-0.3911105053,1.7182225324
C,0,-2.4963310308,-1.6470421459,1.1580075927
C,0,-3.8901944415,-1.9097815773,1.0878506328
N,0,-4.7937475362,-0.9577841908,1.5351618822
C,0,-4.3376805514,0.1899996686,2.0627975474
C,0,-3.0063110097,0.5046852963,2.1581977673
C,0,-1.6251556733,-2.6384711459,0.6489469936
C,0,-2.0843250632,-3.821153391,0.1218903841
C,0,-3.4669857456,-4.0513711676,0.0874014447
C,0,-4.367359017,-3.1243905558,0.5542758871
H,0,4.5464846596,4.3233792416,3.3180329585
H,0,4.0377023938,1.9925212371,2.7166514424
H,0,0.3675322244,5.3491403797,3.2308806959
H,0,2.7431264912,5.9982471858,3.5838859692
H,0,0.032604765,-0.9868581122,1.6115370717
H,0,-5.0959486544,0.8719694552,2.4306199157
H,0,-2.7763815223,1.4374945195,2.6487944124
H,0,-0.5568012027,-2.4681379398,0.6382249074
H,0,-1.3975308856,-4.5610967724,-0.2704767418
H,0,-5.4247457284,-3.3416656224,0.4982897459
C,0,-6.2488466349,-1.2196616683,1.5542411568
H,0,-6.5273821583,-1.6612924759,0.5961412579
H,0,-6.7377519863,-0.2460041381,1.6063319665
C,0,2.2852402795,-0.0526862166,2.0780031824
H,0,1.9126000006,-0.6538708902,1.2466066216
H,0,3.2585279516,0.3281543067,1.7657871657
C,0,-6.6515315685,-2.0886618055,2.73794152
H,0,-6.4023800591,-1.588232758,3.6762501724
H,0,-7.7293991579,-2.2582533154,2.7091340195
H,0,-6.1488609014,-3.0579595986,2.7172521672
C,0,4.9852032507,2.9972440842,-1.8573147118
H,0,6.0704425673,3.046092438,-1.7513741936
H,0,4.7471687826,2.3723957901,-2.720728366
H,0,4.6111143499,4.0058198137,-2.0449595586
C,0,2.3941973266,-0.857606352,3.3661293289
H,0,3.0598400572,-1.7085918697,3.2119982573
H,0,2.7991258657,-0.2375486659,4.1692542943

H,0,1.4147943282,-1.2314342187,3.6739638471
C,0,-4.4898812336,2.8941720775,-1.9284629727
H,0,-5.0536881106,2.4127441299,-2.7310150859
H,0,-5.074622793,3.7324044485,-1.5456142939
H,0,-3.556251717,3.2810322563,-2.344377666
Cl,0,-4.0572166432,-5.5326566779,-0.5987134821
Cl,0,2.5889776031,7.2568572192,0.4972974747
E_{el} = -3559.2597325
G = -3558.598959
Nimag =0

H3

2 1
C,0,-6.8600992239,-0.8601326184,-1.6022582113
C,0,-6.2309277258,0.2360229009,-1.0246123515
C,0,-4.8453734743,0.3387457204,-1.1561776658
C,0,-4.1299544185,-0.6293176553,-1.8649444085
C,0,-4.7581606664,-1.7323900168,-2.4338098765
C,0,-6.1376071978,-1.8381840773,-2.2931952433
N,0,-4.0185191823,1.3390541319,-0.6385640716
C,0,-2.7006706399,1.2242133214,-0.958089608
S,0,-2.431580858,-0.2151254296,-1.9184944546
C,0,-1.7391100341,2.1632904646,-0.5831356866
C,0,-0.3416658089,2.1031285429,-0.7435218706
C,0,0.4921122172,3.240936941,-0.3609755509
C,0,1.9060698054,3.1688115077,-0.4692184719
N,0,2.5053670531,2.0130025333,-0.9453821478
C,0,1.735705805,0.9733692282,-1.3074254161
C,0,0.3679997659,0.9766772603,-1.2204177775
C,0,-0.0570001668,4.4466789317,0.1339994679
C,0,0.7232176557,5.5098529053,0.521860233
C,0,2.1166927503,5.3976421515,0.4102063825
C,0,2.7126787879,4.2595600155,-0.080672588
H,0,-7.9356014989,-0.9582522886,-1.5042407867
H,0,-6.8084015837,0.9821338213,-0.4916757656
H,0,-4.1883961122,-2.4781549918,-2.9768677234
H,0,-6.6552434109,-2.6856053564,-2.7277735062
H,0,-2.1449954705,3.04492008,-0.1141285609
H,0,2.2657266229,0.0995204662,-1.6687426729
H,0,-0.1184040243,0.052640015,-1.4877387795
H,0,-1.1293648601,4.5619222336,0.217605494
H,0,0.2766299476,6.4208124324,0.9013135152
H,0,3.7905256707,4.2225648257,-0.1631858253
C,0,3.963620355,1.9263296552,-1.1695616239
H,0,4.2069146083,0.8626669769,-1.1970001509
C,0,-4.587746686,2.4915776257,0.071991311
H,0,-3.8601122227,2.830820541,0.8091330518
H,0,-5.4433744213,2.1192420722,0.6395506301
H,0,4.4724893283,2.3406268731,-0.2978343295
C,0,-6.5984969972,-2.2565748255,1.4272395445
C,0,-5.2669171267,-2.4664177604,1.0897299199
C,0,-4.3300889826,-1.5192921152,1.5056225693
C,0,-4.7321859595,-0.4094869573,2.2525816677
C,0,-6.0665455911,-0.1935265913,2.5810734328
C,0,-6.9998957823,-1.1332794804,2.1573751814
N,0,-2.9550669537,-1.5242883052,1.2576249843
C,0,-2.2606693758,-0.4997178479,1.8233547194
S,0,-3.3484711638,0.569600696,2.6833913844
C,0,-0.8734290573,-0.3770285652,1.7359784281
C,0,-0.0556803993,0.684979581,2.1667158921
C,0,1.3986071251,0.581853684,2.0636933168
C,0,2.2285553622,1.6685638967,2.4463644333
N,0,1.6595617315,2.8380978536,2.9261573456
C,0,0.32323762,2.9255467674,3.0305517172
C,0,-0.5323993118,1.9168925752,2.6718299364
C,0,2.0405718699,-0.5817483535,1.579705505
C,0,3.406282026,-0.6746211029,1.4542662664
C,0,4.1927108541,0.4231336268,1.8327943831
C,0,3.6317439597,1.576995511,2.3280798224
H,0,-7.3401436342,-2.9799005279,1.1067724087
H,0,-4.9763742433,-3.3422890703,0.5217369679
H,0,-6.363786007,0.6755148166,3.1573773188
H,0,-8.0469205996,-0.9931317633,2.400328686
H,0,-0.3853720304,-1.2219458632,1.2778470288
H,0,-0.0579936484,3.8667451599,3.4098827445
H,0,-1.5826284311,2.146224069,2.7513803491
H,0,1.4563608589,-1.4458872614,1.2930051905
H,0,3.8720559227,-1.5767775479,1.0768536789
H,0,4.2777245666,2.3934139417,2.6215106636
C,0,2.4838393356,3.9568158179,3.4294232353
H,0,1.8452775679,4.8417589725,3.4085206018
H,0,3.2916686452,4.1378555876,2.7186914737
C,0,-2.3223327165,-2.6482054602,0.5549828827
H,0,-3.0320329435,-2.9832268865,-0.2045913983
H,0,-1.4565235621,-2.2675033683,0.0133774071
C,0,2.9991922589,3.6912604916,4.8368467379
H,0,3.6205224813,2.7937595886,4.8756141217
H,0,3.599801735,4.5411863674,5.1659514094
H,0,2.1636241269,3.5653532384,5.528676692
C,0,4.3788310084,2.6126735996,-2.4631920184
H,0,5.4596262831,2.5230132697,-2.5867114651
H,0,3.8893040432,2.1394497019,-3.3170527225
H,0,4.1187564982,3.6735197333,-2.4577548153
C,0,-1.9495302331,-3.7681825625,1.5170171482
H,0,-1.4946277527,-4.5915400884,0.9634847499
H,0,-1.2354468587,-3.4135397644,2.2645438467
H,0,-2.8359877319,-4.1433594331,2.034062713
C,0,-4.9856467675,3.6003434958,-0.8929750931
H,0,-5.7274086765,3.239877966,-1.6097383522
H,0,-5.4159713596,4.4347380073,-0.3363256448
H,0,-4.1151019629,3.9618095494,-1.4461310784
Cl,0,5.9175328366,0.3268188307,1.6570939015
Cl,0,3.1141461771,6.7248679549,0.9188570304
E_{el} = -3559.2581962
G = -3558.597691
Nimag =0

H4

2 1
C,0,-5.3029383199,-1.7535044491,-2.7484909729
C,0,-5.0705105061,-0.6015679045,-2.0062131731
C,0,-3.7559404779,-0.1401000584,-1.9072665804

C,0,-2.7196221735,-0.8120040208,-2.5609878367
C,0,-2.9504201267,-1.9713217532,-3.2944813991
C,0,-4.2580613024,-2.43713074,-3.3816030045
N,0,-3.3021938212,0.9715353349,-1.1969979371
C,0,-1.9695580622,1.2228022224,-1.3019882909
S,0,-1.1955084291,-0.0002656636,-2.2887474943
C,0,-1.36395118,2.3423315753,-0.7324817881
C,0,0.0095467897,2.6392645726,-0.6235727941
C,0,0.4435751497,3.9370898332,-0.1145872027
C,0,1.8246461733,4.2167300778,0.0601776102
N,0,2.7704660106,3.2590752107,-0.2709712545
C,0,2.3678808403,2.0728947566,-0.7515959027
C,0,1.0503645142,1.738047546,-0.9382333358
C,0,-0.4723932454,4.9646305261,0.2124836235
C,0,-0.0686053558,6.1773627044,0.7170861539
C,0,1.3024954325,6.4061630224,0.9039121543
C,0,2.244333342,5.4595970321,0.5776407439
H,0,-6.3184200484,-2.12454863,-2.8376733523
H,0,-5.893269074,-0.0830868713,-1.5272491858
H,0,-2.1352699432,-2.4892897838,-3.78673118
H,0,-4.4684298534,-3.3356174328,-3.9508366071
H,0,-2.0593225284,3.062839149,-0.3316352721
H,0,3.1568205068,1.3665675456,-0.9834918776
H,0,0.8620278853,0.7317187215,-1.2782786192
H,0,-1.533289533,4.8176929517,0.0569647698
H,0,-0.7892284342,6.9483841666,0.9602077031
H,0,3.2915870748,5.684667582,0.723974842
C,0,4.2192712037,3.5435752481,-0.206605298
H,0,4.7200884731,2.5750414661,-0.1843388144
C,0,-4.223049457,1.8309545645,-0.4462726073
H,0,-3.7013893319,2.1697312215,0.4522738647
H,0,-5.0435871021,1.1981095478,-0.1046644628
H,0,4.432973786,4.024867162,0.7484534047
C,0,3.431926887,2.7207965239,3.0362297664
C,0,2.0465822164,2.8334812516,3.042298954
C,0,1.2992203951,1.7495102554,2.5761884202
C,0,1.9374366373,0.5832689454,2.1455852594
C,0,3.3241856195,0.474831974,2.1285394617
C,0,4.0679052622,1.5602087008,2.5796778291
N,0,-0.0887870056,1.6643870659,2.470643365
C,0,-0.5644496777,0.4728876521,2.0178176894
S,0,0.7613076379,-0.610622752,1.6476239157
C,0,-1.9260710256,0.1868490837,1.9329751898
C,0,-2.5590438486,-0.9290892798,1.3491474135
C,0,-4.0005590056,-1.111565538,1.4903672483
C,0,-4.6571242917,-2.1904270028,0.8416786065
N,0,-3.9228673088,-3.0893973821,0.0842538062
C,0,-2.5949630931,-2.9305917728,-0.0247911085
C,0,-1.901883458,-1.907097994,0.5709821338
C,0,-4.7977338631,-0.2452099426,2.2751476112
C,0,-6.1589421963,-0.3967269249,2.38666849
C,0,-6.7765873075,-1.4562491794,1.7060829178
C,0,-6.0538573698,-2.3506702644,0.953127568
H,0,4.027891234,3.5525521931,3.3964135463
H,0,1.5717971614,3.73828431,3.4043543439
H,0,3.8063610262,-0.4312344249,1.7798184361
H,0,5.150541327,1.5020839278,2.5822354822
H,0,-2.5612894697,0.9352198127,2.3795753451
H,0,-2.0817480803,-3.6659164652,-0.633806293
H,0,-0.8458161325,-1.8621744901,0.3552040884
H,0,-4.3395664566,0.5655405479,2.8265896046
H,0,-6.7476736143,0.2799064544,2.9936406583
H,0,-6.5697855121,-3.1619850005,0.4589515914
C,0,-4.5445645706,-4.2844806467,-0.5238154591
H,0,-3.8667262254,-4.6135115184,-1.3123998083
H,0,-5.4680266324,-3.979141649,-1.0168404514
C,0,-0.9589387696,2.769087071,2.8856521873
H,0,-0.4029249507,3.6931904894,2.7206283772
H,0,-1.8088901599,2.7950644096,2.1995729497
C,0,-4.7658156324,-5.3877977621,0.5017983514
H,0,-5.4247462693,-5.0622968967,1.309797927
H,0,-5.2225630735,-6.2489117332,0.0105341254
H,0,-3.8129459987,-5.6988648157,0.935523936
C,0,4.6848464684,4.3708713224,-1.3969956637
H,0,5.7570541347,4.5555724414,-1.3089414437
H,0,4.4994928854,3.8320881631,-2.3288184926
H,0,4.1722196541,5.3340903488,-1.4457983797
C,0,-1.3937170055,2.6371733205,4.3395934733
H,0,-2.039739508,3.475382562,4.6062333458
H,0,-1.9424123291,1.7068150723,4.5053779028
H,0,-0.5223033362,2.6449958523,4.9985450406
C,0,-4.734380298,2.9923771246,-1.2889822825
H,0,-5.2731835955,2.6185421581,-2.1628084446
H,0,-5.4156700444,3.6039420622,-0.6949510128
H,0,-3.9116881741,3.6225770283,-1.6354305737
Cl,0,-8.4977548437,-1.6499481941,1.8337081199
Cl,0,1.8217885208,7.9321873213,1.5499742586
E_{el} = -3559.2573363
G = -3558.598608
Nimag =0

J1

2 1
C,0,-1.1529773398,3.4084685754,-0.2334614817
C,0,-0.3753622871,4.3978029042,0.357845138
C,0,0.994625902,4.1607608977,0.4970032311
C,0,1.5568451338,2.9743767653,0.0199524304
C,0,0.7788967846,1.9873726874,-0.5778238156
C,0,-0.5880075421,2.2128161882,-0.6932521755
N,0,1.9411961048,4.9943377127,1.0926759865
C,0,3.2182815173,4.5321357887,1.0655917794
S,0,3.2828657287,2.9545865496,0.2997290694
C,0,4.3083747961,5.2800128704,1.5046784492
C,0,5.6310423759,4.8384063809,1.7000652924
C,0,6.7134255332,5.7975486043,1.8771594153
C,0,8.0332735332,5.3504932002,2.1452606022
N,0,8.2942059031,3.9922252564,2.2361954819
C,0,7.296921495,3.1111724391,2.0535958026
C,0,6.0032131735,3.478832398,1.7881927677
C,0,6.5072518971,7.1905688831,1.7576494047
C,0,7.5242514973,8.0994799423,1.9228809291
C,0,8.8108677402,7.6231026948,2.2127722302
C,0,9.08047766,6.2790235706,2.3177774459

H,0,-2.2190485932,3.5743697989,-0.3442035076
H,0,-0.8270071154,5.325508496,0.690775914
H,0,1.2325277032,1.0728795487,-0.9445458705
H,0,-1.2181551649,1.4557397082,-1.1470626789
H,0,4.0924682399,6.3159483186,1.7168735684
H,0,7.5696376552,2.0668813982,2.1498626142
H,0,5.2746174382,2.6836239732,1.7307768153
H,0,5.5254497729,7.569906337,1.5062484294
H,0,7.3457799389,9.162915339,1.8217500485
H,0,10.0917974669,5.9587081958,2.5252696352
C,0,9.65984628,3.4791516591,2.4695765041
H,0,9.5459082344,2.4638506854,2.8495607586
C,0,1.5770542665,6.3046648753,1.6418767384
H,0,2.2308484732,6.4993880797,2.4933200424
H,0,0.5673172061,6.2126931285,2.0459050721
H,0,10.1138819967,4.0656318813,3.2691413969
C,0,2.3914635026,4.4788539517,4.6218531831
C,0,1.0752378773,4.2072118326,4.2656766019
C,0,0.8010036761,2.9840827465,3.6482655572
C,0,1.8242259822,2.0577969013,3.4334019384
C,0,3.140373773,2.3276372522,3.7962608637
C,0,3.4170479411,3.5550429941,4.3874407875
N,0,-0.4315680142,2.5316283067,3.1774209087
C,0,-0.424291302,1.2823252445,2.6439680493
S,0,1.1974776933,0.6127208272,2.6739752871
C,0,-1.58055336,0.6226842529,2.2337092142
C,0,-1.6722170072,-0.576269138,1.5010565992
C,0,-2.9388055181,-1.2898293608,1.4040456508
C,0,-3.0458644804,-2.4590457634,0.6070248699
N,0,-1.9370942944,-2.9262128498,-0.0811564069
C,0,-0.7668403854,-2.2756755354,0.0259975472
C,0,-0.5977282172,-1.1446189681,0.7819090737
C,0,-4.0883380866,-0.8760969434,2.1144700156
C,0,-5.284965539,-1.5455745195,2.0273908593
C,0,-5.3612096393,-2.6822694792,1.2097871507
C,0,-4.2720928921,-3.1489694358,0.5124574306
H,0,2.6222429834,5.4260320609,5.0971314711
H,0,0.2908246197,4.9265447511,4.4723649267
H,0,3.9221762255,1.5957840097,3.6238097308
H,0,4.4361047733,3.7939398285,4.6714189261
H,0,-2.5034139807,1.1043539164,2.5185295277
H,0,0.054370864,-2.6892746187,-0.547351234
H,0,0.371064967,-0.6695341048,0.7346432831
H,0,-4.0355777634,-0.0173850006,2.7708721087
H,0,-6.1527507193,-1.2146335248,2.5845281144
H,0,-4.3746793845,-4.0397057398,-0.091269734
C,0,-1.9895866434,-4.1631874617,-0.8872990067
H,0,-1.1518145344,-4.1124887436,-1.5828347782
H,0,-2.8995403884,-4.1418762931,-1.4880808907
C,0,-1.6518539504,3.332834953,3.3193894426
H,0,-1.359044333,4.3803331741,3.2284110564
H,0,-2.2900981268,3.1177943686,2.4610583633
C,0,-1.89234925,-5.4110158978,-0.0194368792
H,0,-2.7067834638,-5.4656386673,0.7061413898
H,0,-1.9378422434,-6.2968921236,-0.6556441999
H,0,-0.9445597499,-5.420525925,0.5232255994
C,0,10.4941320473,3.4852831775,1.195349165
H,0,11.4920758584,3.1019337037,1.4161508932
H,0,10.0343216828,2.8443290595,0.4399506369
H,0,10.5950896688,4.4906243777,0.7807987423
C,0,-2.3513745151,3.0638633858,4.6456742743
H,0,-3.2528916023,3.6750076968,4.7166656816
H,0,-2.635523842,2.0119736874,4.732348651
H,0,-1.6951253155,3.3127578287,5.4831849414
C,0,1.6561462516,7.3998636397,0.5859321933
H,0,0.9722054121,7.1874329101,-0.2393817996
H,0,1.379784902,8.3584266564,1.0287048822
H,0,2.6682684075,7.4815314488,0.1813812885
Cl,0,-6.8716707928,-3.5337000913,1.0902139746
Cl,0,10.1031499908,8.7653378694,2.4273609732
Eel = -3559.2438902
G = -3558.589557
Nimag =0

J2

2 1
C,0,-6.7479825525,-1.2523490272,-1.9526685553
C,0,-6.0677224893,-0.1107013609,-1.5442191396
C,0,-4.6725049997,-0.1419796704,-1.5537884743
C,0,-3.9924054357,-1.2805903475,-1.996789343
C,0,-4.6737287025,-2.4230177218,-2.4033083162
C,0,-6.0644570394,-2.3992977358,-2.3700029682
N,0,-3.803732218,0.8709540307,-1.1479494094
C,0,-2.4824667495,0.6014092759,-1.308092051
S,0,-2.2623295872,-1.0131135976,-1.9601021689
C,0,-1.4826870995,1.5402243562,-1.060939759
C,0,-0.0909347455,1.3389640291,-1.0167323166
C,0,0.8172388851,2.4790763626,-0.9709157765
C,0,2.21639355,2.2798890107,-0.8410400553
N,0,2.72431659,0.9922904337,-0.7675065902
C,0,1.887515173,-0.0555730306,-0.8416358871
C,0,0.5286100322,0.0700975748,-0.9683725126
C,0,0.3564373873,3.8105565978,-1.0808783854
C,0,1.2068871316,4.8886963273,-1.0328799384
C,0,2.5802308922,4.6549626458,-0.875307364
C,0,3.0936539952,3.38262156,-0.7878263309
H,0,-7.8322798523,-1.248992974,-1.9450843305
H,0,-6.6120699649,0.7718406726,-1.2298093059
H,0,-4.1352797408,-3.3056148522,-2.732185919
H,0,-6.6209341707,-3.2780992679,-2.6782764089
H,0,-1.844739062,2.5387424135,-0.8716143832
H,0,2.3515331808,-1.0325430083,-0.7753235477
H,0,-0.0429679641,-0.8452712119,-0.9567425834
H,0,-0.6970738825,4.0062311843,-1.2307272037
H,0,0.8319381118,5.9006643849,-1.1239913467
H,0,4.1615981708,3.2506449692,-0.6856123728
C,0,4.1763422717,0.7354368848,-0.67788391
H,0,4.2861391463,-0.2737373902,-0.2806567703
C,0,-4.306245653,2.1435602568,-0.6177859801
H,0,-3.5942647984,2.4937530629,0.1310939792
H,0,-5.2307354803,1.9196541612,-0.0822827611
H,0,4.5989586988,1.4137189827,0.0640884187
C,0,-1.6529484868,0.1642229102,2.108696999

C,0,-1.5894537325,-1.1304037061,1.6080140619
C,0,-2.7905635721,-1.7956220224,1.3477688756
C,0,-4.0121282053,-1.177080921,1.6252299307
C,0,-4.0756097045,0.1150312649,2.137766548
C,0,-2.8796056172,0.7862211854,2.3688853317
N,0,-2.9537375658,-3.0667915995,0.7977124343
C,0,-4.24555912,-3.4810697002,0.668799672
S,0,-5.3429514976,-2.2430014068,1.2384797301
C,0,-4.5966637099,-4.6895234491,0.075451495
C,0,-5.8904531348,-5.1576888308,-0.2329840669
C,0,-6.0699120329,-6.2223161692,-1.2127769825
C,0,-7.3686847881,-6.7067680248,-1.5184053735
N,0,-8.474834765,-6.1453863398,-0.8999630796
C,0,-8.3047487466,-5.1689504837,0.0044245864
C,0,-7.0767254976,-4.6642817669,0.3507131394
C,0,-4.9831560565,-6.7855108634,-1.9206962122
C,0,-5.1481256912,-7.7935602544,-2.8394074319
C,0,-6.4424043904,-8.2720130681,-3.0886688918
C,0,-7.5433137864,-7.7460767558,-2.4554615321
H,0,-0.7284107072,0.6980973695,2.3004703459
H,0,-0.6290285377,-1.6019938499,1.4356570829
H,0,-5.0321252617,0.5809493001,2.348879872
H,0,-2.9004497018,1.7962387308,2.762841779
H,0,-3.7635222455,-5.2926796116,-0.2492028263
H,0,-9.209452856,-4.8005350229,0.4736780721
H,0,-7.0684379844,-3.9318201588,1.1430193764
H,0,-3.9814183217,-6.4098613503,-1.7605790399
H,0,-4.3008239772,-8.2091436028,-3.3707856072
H,0,-8.5228003549,-8.1408498425,-2.6856469484
C,0,-9.842039709,-6.645935251,-1.1537811383
H,0,-9.9648427493,-6.7747635448,-2.2297436969
H,0,-10.5213382672,-5.8485855887,-0.8528291828
C,0,-1.8107951952,-3.8812307082,0.3716625204
H,0,-2.0654192289,-4.344535557,-0.5843432333
H,0,-0.9891999544,-3.1945921435,0.16606434
C,0,-10.1319992776,-7.9220597327,-0.3750187744
H,0,-10.0492998912,-7.7361627094,0.6979961849
H,0,-11.1487655667,-8.2537347121,-0.5933108467
H,0,-9.4425549699,-8.7261567429,-0.6409304386
C,0,4.8589662637,0.8560732508,-2.0339946233
H,0,5.9268378669,0.661378519,-1.9185672285
H,0,4.4453299503,0.1243664401,-2.731423632
H,0,4.7347265292,1.8523560176,-2.4638361466
C,0,-1.4221238862,-4.9108674098,1.4241816859
H,0,-0.5728138399,-5.4975170403,1.0693877399
H,0,-1.1397983669,-4.4149187035,2.3557119741
H,0,-2.2524698404,-5.5904367587,1.6309621019
C,0,-4.5341804487,3.166544458,-1.7231274984
H,0,-5.2609957741,2.7934513886,-2.4487576523
H,0,-4.9172069435,4.0934402272,-1.2923705944
H,0,-3.6028600716,3.3869930201,-2.2509822457
Cl,0,-6.6601255027,-9.553640476,-4.2418037943
Cl,0,3.6635733535,6.0123850056,-0.8063125829
E_{el} = -3559.2426261
G = -3558.588662
Nimag =0

J3
2 1
C,0,-7.2982510566,0.2306161603,-1.6722951729
C,0,-6.5090729407,1.1388479865,-0.9757525127
C,0,-5.1249515735,1.0724282775,-1.1493566691
C,0,-4.5667005178,0.1260380154,-2.0119734515
C,0,-5.3557992557,-0.7860342635,-2.7033266054
C,0,-6.7335315403,-0.7247287482,-2.5234327205
N,0,-4.1567373254,1.8672435378,-0.5339948129
C,0,-2.872863251,1.6222384382,-0.9236085552
S,0,-2.825810641,0.2923413987,-2.0656652193
C,0,-1.7953271687,2.3993955123,-0.513070498
C,0,-0.4259156106,2.2172298857,-0.8024001486
C,0,0.5276721708,3.2904180394,-0.5509470934
C,0,1.9097740127,3.0993760724,-0.813080978
N,0,2.3535867177,1.8860440499,-1.3143233544
C,0,1.4711684529,0.9026034777,-1.5480396048
C,0,0.1246208684,1.0241386306,-1.3156015006
C,0,0.1269419788,4.5585401256,-0.0704488094
C,0,1.0238623595,5.5711446751,0.1678921786
C,0,2.3841585323,5.3383587039,-0.0811033271
C,0,2.8353755185,4.1350387878,-0.5682521195
H,0,-8.3747494485,0.2621353266,-1.541753163
H,0,-6.9678758848,1.8778876314,-0.3289287216
H,0,-4.9065812581,-1.5202588517,-3.3626246107
H,0,-7.3736097506,-1.419320536,-3.0559116433
H,0,-2.0648778861,3.2684105663,0.0653087443
H,0,1.8886507586,-0.0236634185,-1.9244046909
H,0,-0.4702930759,0.136988877,-1.4716334379
H,0,-0.9201309099,4.7652081672,0.1052771091
H,0,0.693059072,6.5357939978,0.5325045451
H,0,3.8915510082,4.0058122888,-0.7581487736
C,0,3.7732276238,1.6620365097,-1.6585098652
H,0,3.9151726746,0.5817750796,-1.6879377997
C,0,-4.5356197843,2.9969051372,0.3219597591
H,0,-3.7608819128,3.1272556613,1.0787047883
H,0,-5.4435907138,2.7035671223,0.852631962
H,0,4.3874530978,2.0391611954,-0.8403074074
C,0,-13.4683303224,-5.2137091993,-0.2687967653
C,0,-12.206145802,-5.0440541707,-0.8251181693
C,0,-11.3190039013,-4.1785506039,-0.183130977
C,0,-11.6950889601,-3.5229848351,0.991590154
C,0,-12.9601857132,-3.6877254467,1.5444492629
C,0,-13.8464811177,-4.5431440608,0.8993516739
N,0,-10.0215048905,-3.8532770722,-0.580081195
C,0,-9.3481694023,-3.0276736525,0.2616102773
S,0,-10.3803460459,-2.5350512386,1.5906737709
C,0,-7.9972920875,-2.719318074,0.1024092881
C,0,-7.2265111369,-1.8020728675,0.8381792595
C,0,-5.7712102008,-1.8177962419,0.7484373741
C,0,-4.9968018796,-0.8816581174,1.4823294296
N,0,-5.6277418426,0.0625115327,2.2780460302
C,0,-6.9723673509,0.0803129589,2.3480791498
C,0,-7.7739928702,-0.7966163913,1.668564542
C,0,-5.0740118817,-2.7715697385,-0.02731962
C,0,-3.7012611426,-2.8027198857,-0.0978709527

C,0,-2.9711208551,-1.8540049688,0.6303499562
C,0,-3.5884756098,-0.9035583526,1.4096839499
H,0,-14.1700234846,-5.8835648144,-0.7528873874
H,0,-11.9248375843,-5.5784561183,-1.7247800874
H,0,-13.24095741,-3.1677010862,2.4531848575
H,0,-14.8379247807,-4.6938167608,1.3107462939
H,0,-7.4942655495,-3.2920194023,-0.6611758213
H,0,-7.3982476651,0.8541140247,2.9762975128
H,0,-8.8387674424,-0.6316547866,1.7402016797
H,0,-5.6216673916,-3.5222667548,-0.5819211751
H,0,-3.188563294,-3.5459301757,-0.6960924848
H,0,-2.9791596219,-0.2003346493,1.9609823707
C,0,-4.8623057484,0.9519171488,3.1753427864
H,0,-5.5211129127,1.7851134944,3.4229872735
H,0,-4.0207872659,1.3662330262,2.6189491175
C,0,-9.4269368614,-4.4212741095,-1.7952591421
H,0,-10.2350290096,-4.5383927285,-2.5186486412
H,0,-8.7452094862,-3.6775648881,-2.2095318189
C,0,-4.4106562212,0.2269750111,4.4359844303
H,0,-3.7662026133,-0.6229889086,4.2010651101
H,0,-3.8529684819,0.9186612888,5.0699576411
H,0,-5.2770369458,-0.1363000152,4.9929603986
C,0,4.1378492169,2.2881503888,-2.9982902984
H,0,5.1919361821,2.097919202,-3.2085787244
H,0,3.5385507918,1.8471398886,-3.7977738294
H,0,3.975930596,3.3681852369,-2.99990128
C,0,-8.7299449153,-5.7470915509,-1.5171822051
H,0,-8.3071389787,-6.1420092398,-2.4426938668
H,0,-7.9226641482,-5.6204628416,-0.7913588493
H,0,-9.438759345,-6.4772929998,-1.1191713529
C,0,-4.7564066465,4.2681135436,-0.4881591314
H,0,-5.5516457753,4.1196034794,-1.2225006608
H,0,-5.0436602453,5.0839413222,0.1774222894
H,0,-3.8443476865,4.5539683827,-1.0179402172
Cl,0,-1.2351124492,-1.8782698734,0.5517757206
Cl,0,3.526114306,6.6131710044,0.2206174922
E_{el} = -3559.240024
G = -3558.586982
Nimag =0

J4

2 1
C,0,-5.0713994743,-0.7326658966,-2.7334240991
C,0,-4.6782001409,0.2809667048,-1.8685434756
C,0,-3.3162937505,0.5737700375,-1.7820223994
C,0,-2.3888515995,-0.1262742852,-2.5568375614
C,0,-2.7822113307,-1.1453054072,-3.41780628
C,0,-4.1379934037,-1.44155876,-3.4980840251
N,0,-2.710528925,1.5322020611,-0.9683004496
C,0,-1.3691952913,1.6506948489,-1.1156249053
S,0,-0.770438631,0.4708186144,-2.2636106728
C,0,-0.619576058,2.6567796685,-0.4953718201
C,0,0.7802659073,2.7931799547,-0.4756810432
C,0,1.3986862511,4.0543669408,-0.0840681885
C,0,2.8114938284,4.1613310191,0.0109006429
N,0,3.606539168,3.0649577145,-0.2820376624
C,0,3.0336884153,1.9098728807,-0.6627487204
C,0,1.6784110313,1.7406501204,-0.7702530694
C,0,0.6353710159,5.2093637439,0.1990915909
C,0,1.2149554104,6.3923961665,0.5933889011
C,0,2.6110890148,6.4529032036,0.7087537631
C,0,3.4104841287,5.3734657152,0.4129307284
H,0,-6.1250907768,-0.9738904919,-2.8162130063
H,0,-5.412823554,0.8282006638,-1.2898374453
H,0,-2.0524879041,-1.6857237306,-4.0095768137
H,0,-4.4734627793,-2.2280061141,-4.1643117119
H,0,-1.2060218564,3.4370793672,-0.0329066999
H,0,3.7147668575,1.0907755594,-0.8615977345
H,0,1.3335519144,0.7442416244,-1.0055042136
H,0,-0.442065934,5.1848980194,0.0950487167
H,0,0.6103501746,7.2644488083,0.8114684715
H,0,4.4847837624,5.4741376918,0.4965383298
C,0,5.0800106765,3.1429142277,-0.2339602266
H,0,5.4404020411,2.1180127389,-0.1429002945
C,0,-3.4910034219,2.3549326844,-0.0377218824
H,0,-2.8579263622,2.563675572,0.8254786587
H,0,-4.3127519419,1.7344078913,0.3232889476
H,0,5.3620361813,3.6592921846,0.6851477351
C,0,8.8692166236,8.6263750542,3.8569573266
C,0,7.524122278,8.6277888161,4.2055867667
C,0,6.7644278066,7.4983163678,3.8943597235
C,0,7.3606590006,6.402660348,3.2659197802
C,0,8.7064752623,6.3994816757,2.9174753294
C,0,9.4575933493,7.5300318491,3.2173735051
N,0,5.4064543061,7.2935659893,4.1469388269
C,0,4.9287836729,6.0732594551,3.7884761741
S,0,6.1937002827,5.1181187028,3.0432263711
C,0,3.5937439783,5.7046164233,3.9386737623
C,0,2.965534173,4.5096226001,3.5425834516
C,0,1.5107806093,4.4491763811,3.4446673079
C,0,0.8657966776,3.2532702955,3.0360929661
N,0,1.6220155209,2.1329082605,2.7231766595
C,0,2.9608856463,2.1854676948,2.8370397763
C,0,3.6447515737,3.3112396782,3.2191340698
C,0,0.6938065747,5.5742907062,3.7000609805
C,0,-0.6752597551,5.5307845104,3.5918903989
C,0,-1.27911117,4.3275062263,3.2052722057
C,0,-0.5397918108,3.2007108061,2.9251296041
H,0,9.4725737258,9.4958115057,4.0923731817
H,0,7.0915699458,9.4816231263,4.7127589512
H,0,9.1520642926,5.539893211,2.429843632
H,0,10.5096182814,7.557029902,2.9579478604
H,0,2.9673467108,6.4715592209,4.3650572151
H,0,3.4873053339,1.2617627263,2.6234630271
H,0,4.7128506095,3.2039685284,3.33779445
H,0,1.1428638207,6.5219522642,3.9661934056
H,0,-1.2794659065,6.4079390671,3.7877696953
H,0,-1.0476691475,2.2879456188,2.6445379861
C,0,0.9885069085,0.8363718678,2.3986577646
H,0,1.746678724,0.2446924947,1.8843948637
H,0,0.1881720713,1.017530751,1.6776870927
C,0,4.5784267694,8.3210148839,4.7904394421
H,0,5.0289592077,9.2848655167,4.5543632913

H,0,3.6001097428,8.3171630416,4.3065682381
C,0,0.4975503901,0.1189319698,3.6486211674
H,0,-0.2429951635,0.7104399771,4.1913007989
H,0,0.0386759918,-0.8289734686,3.3618021539
H,0,1.3343247837,-0.0878744932,4.3193388849
C,0,5.6571218765,3.8099372457,-1.4750782449
H,0,6.7446876097,3.8478681745,-1.3897104851
H,0,5.3974870944,3.2358952261,-2.3670884216
H,0,5.2849727808,4.8291830044,-1.5986886252
C,0,4.4785175704,8.1034895548,6.2942376156
H,0,3.8614191167,8.8863589258,6.7387465012
H,0,4.0264670546,7.1342871405,6.5187467253
H,0,5.4700738129,8.1386463253,6.7516041366
C,0,-4.0018514438,3.6331250872,-0.6891932567
H,0,-4.6552456421,3.4012674659,-1.5337381545
H,0,-4.5700503494,4.2121081826,0.0415152488
H,0,-3.1751139267,4.248198785,-1.0534965364
Cl,0,-3.0111174405,4.2632838938,3.0707985086
Cl,0,3.3543141408,7.9340390238,1.2332683151
E_{el} = -3559.2473326
G = -3558.591517
Nimag =0

References:

1. Legault, C.Y. CYLview, version 1.0 b; Université de Sherbrooke: Sherbrooke, QC, Canada, 2009.(http://www.cylview.org).
